# Alternative Isoform Analysis of Ttc8 Expression in the Rat Pineal Gland Using a Multi-Platform Sequencing Approach Reveals Neural Regulation

**DOI:** 10.1371/journal.pone.0163590

**Published:** 2016-09-29

**Authors:** Stephen W. Hartley, James C. Mullikin, David C. Klein, Morgan Park, NISC Comparative Sequencing Program, Steven L. Coon

**Affiliations:** 1 Comparative Genomics Analysis Unit, Cancer Genetics and Comparative Genomics Branch, National Human Genome Research Institute, National Institutes of Health, Bethesda, Maryland, 20892, United States of America; 2 Section on Neuroendocrinology, Program in Developmental Endocrinology and Genetics, Eunice Kennedy Shriver National Institute of Child Health and Human Development, National Institutes of Health, Bethesda, Maryland, 20892, United States of America; 3 National Institutes of Health Intramural Sequencing Center, National Human Genome Research Institute, National Institutes of Health, Rockville, Maryland, 20852, United States of America; Florida Atlantic University, UNITED STATES

## Abstract

Alternative isoform regulation (AIR) vastly increases transcriptome diversity and plays an important role in numerous biological processes and pathologies. However, the detection and analysis of isoform-level differential regulation is difficult, particularly in the face of complex and incompletely-annotated transcriptomes. Here we have used Illumina short-read/high-throughput RNA-Seq to identify 55 genes that exhibit neurally-regulated AIR in the pineal gland, and then used two other complementary experimental platforms to further study and characterize the Ttc8 gene, which is involved in Bardet-Biedl syndrome and non-syndromic retinitis pigmentosa. Use of the JunctionSeq analysis tool led to the detection of several novel exons and splice junctions in this gene, including two novel alternative transcription start sites which were found to display disproportionately strong neurally-regulated differential expression in several independent experiments. These high-throughput sequencing results were validated and augmented via targeted qPCR and long-read Pacific Biosciences SMRT sequencing. We confirmed the existence of numerous novel splice junctions and the selective upregulation of the two novel start sites. In addition, we identified more than 20 novel isoforms of the Ttc8 gene that are co-expressed in this tissue. By using information from multiple independent platforms we not only greatly reduce the risk of errors, biases, and artifacts influencing our results, we also are able to characterize the regulation and splicing of the Ttc8 gene more deeply and more precisely than would be possible via any single platform. The hybrid method outlined here represents a powerful strategy in the study of the transcriptome.

## Introduction

Transcriptome complexity is amplified by alternative isoform regulation (AIR), a broad category of regulatory phenomena which can involve alternative splice sites, alternative transcription start sites, intron retentions, exon skipping, methylation, nucleosome occupancy, internal promoters, nonsense mediated decay, and/or transcript switching. Through these changes, proteins encoded by different transcripts of a single gene can have very different biological functions.

This paper describes an initial effort aimed at understanding AIR in the rat pineal gland, the neuroendocrine structure responsible for the 24-hour rhythm in melatonin production [1, 2]. The pineal gland evolved from ancestral photoreceptors that also gave rise to the retina; these two organs share a common genetic pattern and develop from the same primordia [3–13].

The pineal transcriptome undergoes robust neurally-regulated circadian changes [5, 14], driven by the endogenous clock in the suprachiasmatic nuclei (SCN). Signals from the SCN pass through a multi-synaptic pathway that includes the superior cervical ganglia (SCG). At night, norepinephrine is released from the SCG projections to the pineal gland, initiating a chain of events that leads to changes in the transcription of thousands of genes [5, 14]. Accordingly, when the SCG is removed (SCGX) or decentralized (DCN) most of these night/day differences are not observed [14, 15]. The night/day changes can be reproduced in organ culture by treating the pineal gland with norepinephrine or an analog of cyclic AMP, a second messenger for norepinephrine in this tissue [5, 14].

In the literature there is evidence suggesting that neurally-regulated alternative isoform regulation may occur in the rat pineal gland. Firstly: a splicing regulator gene, Mbnl2, is strongly upregulated at night [5, 14, 16]. This gene contains an RNA-binding domain [17] and regulates alternative splicing in humans [18, 19], mice [20], zebrafish [21], and drosophila [22]. In addition, several genes have already been found to exhibit neurally-controlled, rhythmic, isoform-specific differentials in the rat pineal gland, including Crem [23–25], Pde4b [26], Slc15a1/Pept1 [1, 27], and Atp7b [28]. Accordingly, further study might reveal additional differentially regulated alternative isoforms of these and other genes, and provide further insight into the molecular biology of the regulatory mechanisms involved.

Although interest in AIR is high, progress in understanding its functional importance has been slowed by the limitations of next-generation high-throughput RNA sequencing technologies (RNA-Seq). A major factor influencing this progress is the low quality of the extant transcript annotations, which is particularly sparse for the rat transcriptome. The Ensembl transcript annotations (release 80) for rat, mouse, and human all have roughly the same number of protein coding genes (22093, 22114, and 22002, respectively). However, only 22% of known genes have more than one known protein coding transcript in the rat annotation, compared with 60% and 84% in mouse and human annotations, respectively. The sparse annotation of the rat transcriptome presents a substantial obstacle to the detection of alternative isoform regulation, as many of the most popular isoform-level tools (including Kallisto [29], eXpress [30], and RSEM [31]) implicitly assume that the supplied annotation is both correct and comprehensive, and generally cannot assess the expression of unknown isoforms.

While some tools such as CuffLinks [32] can rescue some of these unannotated isoforms, these methods are of limited utility due to the inherent difficulty in assembling long transcripts using short read data [33, 34]. This inherent difficulty has become increasingly obvious in recent years with the development of long-read RNA-Seq. These new technologies, including Pacific Biosciences (PacBio) single-molecule real-time (SMRT) sequencing, provide the unprecedented ability to sequence transcripts across their entire length at high coverage depth. Several recent studies using these technologies have shown that in many cases the existing transcript annotations (which are often at least partially based on high-throughput/short-read assemblies) are highly incomplete, missing dozens or even hundreds of expressed transcripts [33, 35–37].

In this paper we apply a multi-platform “hybrid” methodology to study the alternative isoform regulation of the Tetratricopeptide Repeat Domain 8 gene (Ttc8, also known as Bbs8) in the rat pineal gland. Short-read/high-throughput stranded RNA-Seq data from a recently-published study [14] was analyzed using JunctionSeq [38] to detect, assess, and characterize differentially regulated alternative splice variants across the entire genome. Based on these results the Ttc8 gene was selected for further investigation. The short-read RNA-Seq results were validated and augmented using quantitative PCR (qPCR) and SMRT long-read RNA sequencing.

JunctionSeq is a Bioconductor package that detects “differential usage” (DU) of exons and splice junctions, in which individual “sub-features” (exons and splice junctions) display patterns of expression that differ from the overall pattern of read-coverage across their respective genes. The detection of differential usage of these features is intended to serve as a proxy for the detection of differential alternative isoform regulation. JunctionSeq can query novel splice junctions without knowledge of the full-length isoforms to which they belong, and has been shown to provide powerful detection of differential isoform usage even when provided with incomplete annotations [38]. This trait makes it particularly suited for the analysis of the rat pineal transcriptome.

In this study we identified 55 genes that exhibit neurally-regulated differential usage of exons and splice junctions. We focused on the alternative isoform usage of the Ttc8 gene in part because mutations in Ttc8 have been implicated in various ciliopathies including non-syndromic retinitis pigmentosa (RP) [39, 40] and in Bardet-Biedl syndrome (BBS, which commonly includes RP among its symptoms) [41, 42]. At least one form of non-syndromic RP is caused by defective splicing of a retina-specific splice variant in humans [39, 40]. Given the similarities between the pineal gland and retinal cells, it is possible that an in-depth study of alternative splicing in the pineal gland might also shed light onto its biological role in vision.

Although the exact function of the Ttc8 gene is not well-understood, the gene appears to be involved in protein transport in and out of cilia as part of a large complex called the BBSome [43]. Ttc8 proteins are structurally characterized as containing 8 tetratricopeptide repeats (TPR) in the C-terminal half of the protein, forming an extensive protein-protein interaction domain [44, 45].

Our hybrid approach leverages the complementary strengths of our various experimental platforms, offering new insight into the complex isoform-level expression regulation taking place in the Ttc8 gene. This report provides a valuable template for future investigation of such regulatory phenomena.

## Results

### Detection of differential isoform usage via JunctionSeq

JunctionSeq detected more than 500 genes displaying statistically significant differential exon or splice-junction usage in data from each of two *in vivo* experimental groups (control and sham) comparing day and night (adjusted p-value < 0.05, see Table 1). In each of the two *in vitro* experiments (CN vs NE and CN vs DBcAMP), more than 200 genes showed similar statistical significance. We found strong concordance in the genes detected in each of these four analyses, particularly between the two *in vivo* experiments and the two *in vitro* experiments (see Table 1 and S1 Fig). Even at the (extremely conservative) adjusted p-value threshold of 0.0001, we found 18 genes that showed statistically significant differential usage in all four experiments (see Table 1 and Table 2). Three of these top genes were already known in the literature to exhibit neurally-controlled alternative isoform regulation.

**Table 1:** The number of genes detected in each JunctionSeq analysis at various p-value cutoffs. Also shown is the overlap between the genes detected across multiple separate analyses. Day and night conditions were compared in the four *in vivo* groups: Control (Ctrl), Sham, decentralized (DCN), and ganglionectomized (SCGX). The two *in vitro* analyses compared untreated (Untr) pineal glands with pineal glands treated with norepinephrine (NE) or (Untr) vs DBcAMP (DB).

p-adjust	*In Vivo–*Normal Day vs Night			*In Vitro* Untreated vs Treated			Shared All 4	*In Vivo*–Neural Stimulus Removed	
	Ctrl	Sham	Both	Untr/NE	Untr/DB	Both		DCN	SCGX
**0.05**	620	557	224	219	260	120	55	18	50
**0.01**	394	303	156	140	188	87	40	8	36
**0.001**	263	189	108	86	124	60	28	4	21
**0.0001**	202	136	87	64	88	47	18	2	18
**0.00001**	168	109	75	49	73	38	15	2	12
**0.000001**	142	94	60	42	65	34	14	2	9

**Table 2:** Summary information on the 18 genes detected at p-adjust < 0.0001 in all four analyses. Column 3 indicates the number of distinct exonic regions (exons), known splice junctions (SJ) and novel splice junctions belonging to this gene, and columns 4–7 indicate the number of each feature type with statistically significant differential usage (# sig, p-adjust < 0.0001). The rightmost column indicates the least significant gene-wise adjusted p-value across the four analyses. *Genes previously known in the literature to exhibit differential isoform regulation between night and day in the rat pineal gland: Crem [23–25], Pde4b [26], and Atp7b [28].

Gene Symbol	Locus	# Features (exons/ known SJ/ novel SJ)	# Sig. (exons / known SJ / novel SJ)				Least sig. gene-wise p-adjust
			Ctrl Day/Night	Sham Day/Night	Untr vs NE	Untr vs DB	
Arsg	chr10:97771263-97863311(+)	12/11/3	5/4/1	3/3/1	4/1/0	3/1/0	0
Commd1	chr14:107664254-107760191(-)	3/2/4	1/1/3	1/1/3	3/1/3	2/1/3	0
Crem*	chr17:57031765-57090888(+)	21/15/5	19/8/3	16/6/2	18/9/1	19/9/1	0
Dclk1	chr2:144646307-144939389(+)	21/20/0	18/17/0	18/17/0	16/8/0	11/7/0	0
Ggcx	chr4:100277390-100293250(+)	20/16/1	7/6/0	4/5/1	6/2/0	6/4/0	0
Alox15	chr10:56953690-56962161(-)	14/13/10	0/1/5	0/1/3	0/0/1	0/1/3	4.62E-13
Atp7b*	chr16:74865515-74947084(+)	23/21/2	7/3/0	6/3/0	8/1/0	7/0/0	1.13E-12
Ugp2	chr14:106208175-106249103(-)	13/10/3	1/1/0	1/1/0	1/1/0	1/1/0	2.04E-10
Pde4b*	chr5:121952976-122136814(+)	19/14/1	7/1/1	6/4/1	5/4/1	5/3/1	9.08E-10
**Ttc8**	**chr6:122920316-122977132(+)**	**18/16/6**	**2/1/3**	**2/1/4**	**1/1/1**	**2/1/1**	**7.26E-08**
Tsc22d1	chr15:58554373-58658153(+)	5/3/2	2/0/1	0/0/1	0/0/1	3/1/1	2.24E-07
Eprs	chr13:103300931-103371577(+)	38/35/2	36/26/0	21/17/0	8/5/0	8/5/0	2.62E-07
Map3k5	chr1:15412602-15613746(+)	32/32/0	26/10/0	13/6/0	4/2/0	3/1/0	4.43E-07
Btrc	chr1:265157378-265269837(+)	14/13/1	1/0/0	2/1/0	1/0/0	1/0/0	6.23E-07
Slc38a1	chr7:137975548-138039984(-)	19/16/6	0/0/2	2/0/2	0/0/2	0/0/2	2.11E-06
Arhgap24	chr14:8383315-8600424(-)	10/8/4	0/0/1	1/0/2	0/0/2	3/1/3	1.54E-05
Cbx6	chr7:121050301-121058029(-)	5/4/2	5/2/1	4/2/0	5/3/0	5/3/0	2.86E-05
Pla2g4a	chr13:66849776-67206693(-)	20/18/1	0/0/1	0/0/1	0/0/1	0/0/1	7.00E-05

As expected, we found very few genes that displayed significant differential usage in the two “stimulus deprived” experiments (DCN and SCGX, see Table 1); the DCN experiment provided the fewest. The small number of genes detected in the DCN analysis may represent either false discoveries or real night/day differences caused by factors other than neural stimulation via the SCG.

Of the top 18 genes detected by JunctionSeq as exhibiting differential usage in all four neural stimulus experiments, Ttc8 was selected for further validation and investigation, because several of the statistically-significant splice junctions were novel, the effect was strong and consistent in all four experiments, and the gene had been previously identified in the literature as having an alternatively spliced isoform with a strong phenotypic effect in the retina [39, 40].

The complete JunctionSeq results for all six analyses and all genes that displayed significant differential usage can be found in the S4 Dataset and S5 Dataset.

### Differential transcription start site usage of the Ttc8 Gene

**Fig 1:**
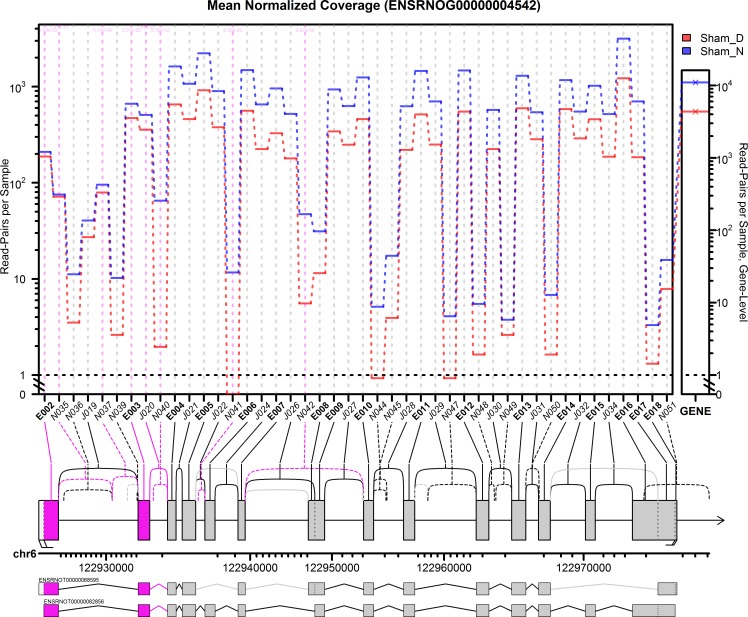
JunctionSeq gene profile plot for Ttc8 gene, sham night/day experiment. This plot displays the estimates for the mean normalized read-pair coverage count for each exon and splice junction. The small plotting panel on the far right displays the total mean normalized read-pair count for the gene as a whole. The gene diagram below the main plotting frame displays the exonic regions (boxes, labelled E001-E018), known splice junctions (solid lines, labelled J019-J034) and novel splice junctions (dashed lines, labelled N035-N051). Significant features are drawn in pink, and features that did not pass the automatically-selected minimum count threshold were colored in light grey (and were not plotted). Beneath the gene diagram, the genomic positions are marked with ticks at each kilobase. Note that the scale is not uniform due to the nonlinear expansion of small features to improve readability. Beneath the scale the two Ensembl known isoforms are drawn. The upper isoform is the RefSeq transcript.

**Fig 2:**
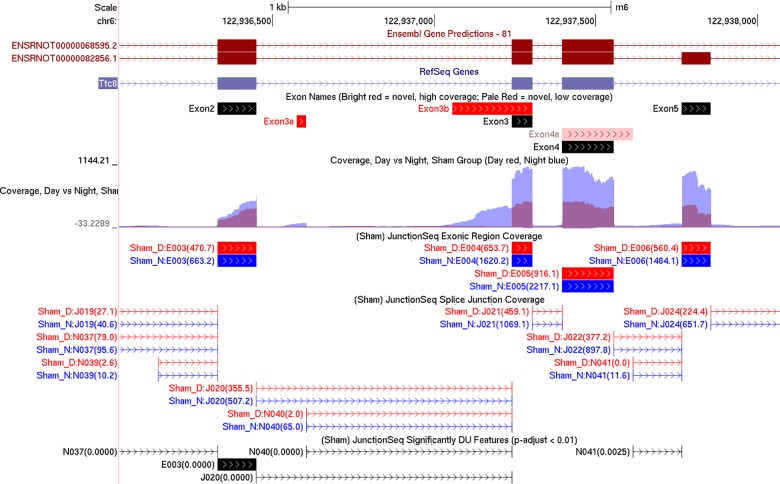
Summary browser tracks generated by QoRTs/JunctionSeq for the region surrounding exons 2 to 5 of Ttc8. The top two tracks display the Ensembl and RefSeq annotations, respectively. The third track displays the names of the exons as they are referred to in the text and in previous literature, with our own names assigned to previously-unidentified exons. The fourth track, generated by QoRTs, displays the mean normalized read-pair coverage depth at each genomic position for night (blue) and day (red) in the sham group. The fifth and sixth tracks, generated by JunctionSeq, display the mean normalized read counts across each known exonic region and across each splice junction (respectively). The bottom track, also generated by JunctionSeq, displays the exons or splice junctions found to display statistically significant differential usage (at p-adjust < 0.01).

In the Ttc8 gene we detected 17 novel splice junctions that surpassed our initial coverage cutoff, all 17 of which used canonical GT-AG dinucleotide donor-acceptor sites. The following transcript annotation databases were checked to determine whether these exons and splice junctions were novel: Ensembl, RefSeq, the Mammalian gene Collection, and RGD. Only two of these novel splice junctions were present in any of these databases (see S13 Fig, S14 Fig and S15 Fig). Based on the gene profile plots (Fig 1 and Fig 2), we can separate the Ttc8 gene into three distinct regions based on their expression. The known 5’ start site displays roughly constant high-level coverage at both night and day. The novel splice junction N040 (which appears to splice from an alternative 5’ start site) displays an extreme differential with very low coverage during the day (~3 read-pairs per sample) and moderate coverage at night (~60 read-pairs per sample). Finally, the remainder of the gene appears to display a composite of these two, with higher overall coverage, but weaker relative night/day differences than the novel junction N040. Note that none of the novel exons or splice junctions appear in any of the transcript annotations. Note that the Rat Genome Database (RGD) is not included in this plot because it has not yet been lifted over to the rn6 rat genome build. See (S15 Fig) for the RGD annotation.

This lends itself to the hypothesis that splice junction N040 is associated with an internal promoter that is expressed almost exclusively at night, but still only at moderate levels. The novel exon associated with this internal promoter will be referred to as “exon 3a”. The annotated 5’ start site, on the other hand, appears to display consistently high expression at both day and night. Examination of the “wiggle” plots for Ttc8 further revealed that there also appears to be an unannotated 5’ transcription start site leading directly into exon 3 (Fig 2). This alternative exon form will be referred to as “exon 3b”. Like exon 3a, this region has almost no read coverage during the day but moderate read coverage at night.

**Fig 3:**
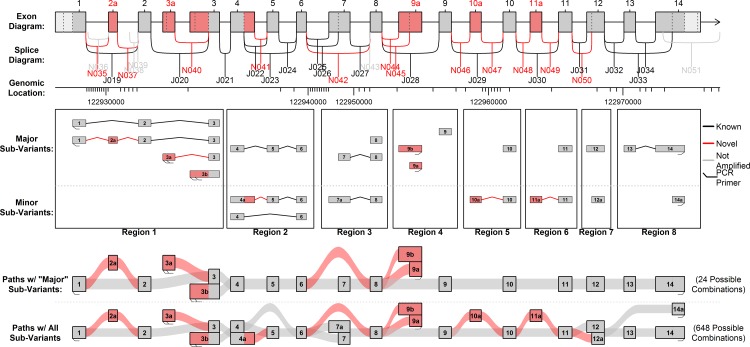
All possible sub-variants detected in the Illumina RNA-Seq data. Considering the three major 5’ ends and two major 3’ ends, there are 21 “sub-variants” across 8 “regions”, which can be combined to form 648 different possible combinations. Based on coverage in the Illumina and PacBio data, we narrowed this down to 9 “major” sub-variants that appear to be expressed in the pineal gland in substantial quantities. There are 24 possible combinations of these 9 major sub-variants.

Evidence of multiple novel splice junctions, transcription start sites, and terminal exons was found in the Illumina RNA-Seq data (see Fig 3). Combinatorially generating all possible combinations of these variable regions (or “sub-variants”) yielded 648 theoretical isoforms (see S2 Dataset). Most of these sub-variants were only detected at very low levels and did not display significant differential usage. Some additional features that appeared to lead to additional alternative start and end sites were not considered in this analysis.

**Fig 4:**
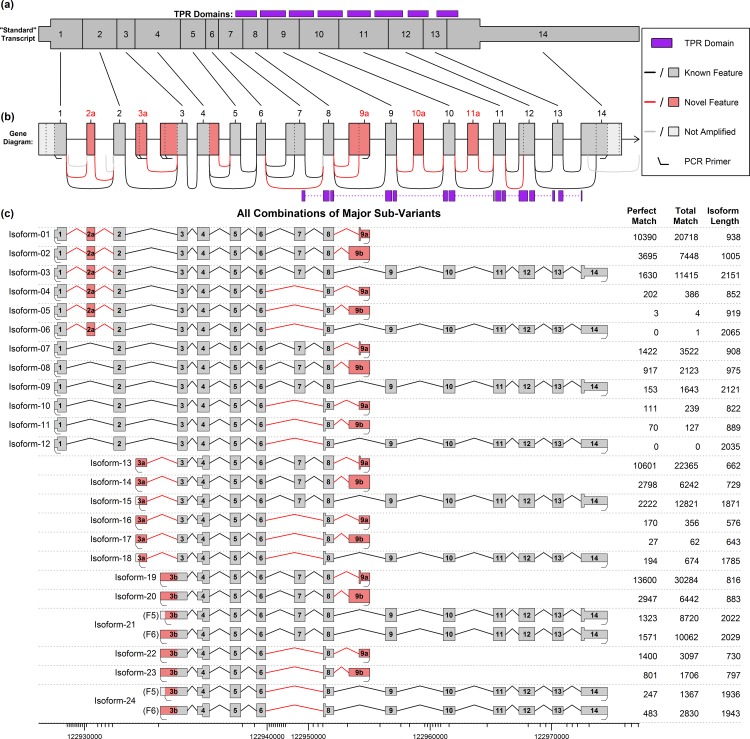
Diagram of the 24 potential isoforms produced by the “major” sub-variants, with PacBio match counts. Part (a) and (b) display the “standard” known isoform and a diagram of the whole gene, respectively. The wide portion of (a) indicates the coding DNA sequence (CDS) of the “standard” transcript, and the purple boxes in (a) and (b) indicate tetratricopeptide repeat (TPR) domains. Part (c) displays the 24 possible combinations formed by the “major” sub-variants from Fig 3. Novel features are displayed in pink, and the widened region in each transcript denotes the largest open reading frame (ORF). The data table on the right side of (c) displays the number of full-length ROI from the SMRT sequencing that were found to match each isoform. The first column indicates the number of perfect, base-for-base, full-length matches, and the second column lists “alignment matches”, in which the RNA-STAR aligner maps the ROI to the isoform. Note that the novel start sites were each covered by two primers that were less than 6 base-pairs apart, producing slightly different amplified molecules. Isoforms 21 and 24 were covered by two different primer pairs used in the PCR amplification. See S3 Dataset for more information on these theoretical isoforms and their PacBio coverage.

For simplicity, a subset of the sub-variants which seemed to have good support in the data was selected, and will be referred to as the “major sub-variants” (see Fig 3, S3 Dataset). There are 24 different “major sub-variant combinations” that can be constructed using only these components (see Fig 4). Twelve use the known start site (exon 1: potential isoforms 1–12), six use one novel start site (exon 3a; potential isoforms 13–18) and six use the other novel start site (exon 3b; potential isoforms 19–24). Note that these potential isoforms represent all possible combinations of this set of start sites and splice junctions, not all of these combinations necessarily exist and we cannot directly determine which do and do not exist based purely on the short-read Illumina datasets.

#### qPCR validation

Although qPCR cannot be used to accurately assess the expression of different loci relative to one another, it is possible to determine the relative expression of a specific exon or splice junction across multiple conditions. Accordingly, qPCR was used to confirm the differential usage of the three predicted start sites in Ttc8: exon 1 (using exon 2 as a proxy), exon 3a, and exon 3b (see Fig 4).

**Fig 5:**
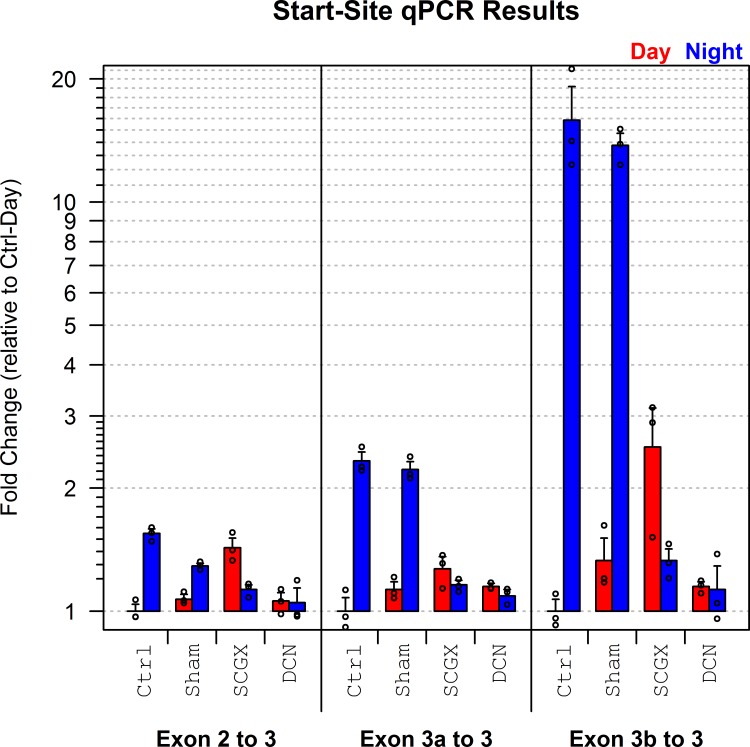
Results of the start-site qPCR experiment. Three primer pairs were used, corresponding to the three transcription start sites detected in the Illumina RNA-Seq data. The novel start sites that begin with exons 3a or 3b both display very strong upregulation at night in the control and sham groups.

All three initial exons displayed a differential increase in expression at night in the neurally stimulated samples (see Fig 5). As expected, both exons 3a and 3b display much stronger differentials than exon 1. In the control group, exon 1 displays a 1.4-fold increase at night, whereas exons 3a and 3b display 2.3-fold and 16-fold increases, respectively. Similar increases in initial exon expression are apparent in the sham group, but not in the SCGX and DCN groups, indicating that the differential usage of these alternative transcription start sites is driven by neural activity.

This confirms the differential expression detected in the four RNA-Seq analyses. Furthermore: the fact that both exon 3a and 3b display substantially stronger differentials than exon 1 confirms the presence of isoform-specific differential usage.

#### Qualitative validation in an independent RNA-Seq dataset

Qualitative examination of a publicly-available RNA-Seq dataset generated from rat pineal glands collected at various times throughout the day [46] revealed that the novel exons 3a and 3b show visible coverage at night but not during the day (see S7 Fig). Hundreds of reads cover these novel regions at ZT19 and ZT23, whereas only a handful of reads cover the same regions at ZT3, ZT7, and ZT15. At the same time the other exons show moderate to high coverage at all time points, indicating that this effect is specific to these alternative initial exons.

Robust statistical analysis of this pattern is not possible because this dataset lacks replicates; however, the simple visual observation that these two novel alternative initial exons are present at night but not during the day is clearly consistent with the previous RNA-Seq results, and thus lend further credence to our conclusions. This dataset also confirms the existence of many of the other alternative splice sites and exonic regions seen in the discovery dataset. For example, we observe exon 2a, the novel skipping of exon 7, and the use of novel alternative terminal exons 9a and 9b.

Similar examination of rat retinal tissue RNA-Seq data over the same time points [46] reveals that there is little-to-no expression of the novel alternative initial exons 3a and 3b in the rat retina at either day or night. The rat retina does include a mixture of inclusion and skipping of exon 2a, and a very small number of reads (less than 10 per sample) appear to splice to exon 9a (see S12 Fig). Almost no reads splice across any of the other splice junctions, which is expected in view of the low coverage depth of these samples.

### Isoform assembly via PacBio SMRT sequencing

Validating differential abundance across these splice junctions does not provide any information about the full-length isoforms to which these junctions belong, and distant variable-splicing regions cannot easily be “phased” using short-read RNA-Seq data. The CuffLinks transcript assembly tool [47] was used on this dataset, but failed to detect any of the novel splice junctions in Ttc8 despite hundreds of reads of coverage across numerous samples (see S1 Dataset).

To validate and identify the Ttc8 isoforms we ran a pooled set of transcript-length PCR-amplified products on a Pacific Biosciences RS II sequencer. This yielded a total of 410,981 “reads of insert” (ROI), which are the consensus sequences collapsed from repeated sub-reads across the same individual molecule (ROI are alternatively called “circular consensus sequence” (CCS) in previous versions of the protocol). Of these, 63% were “full length”, i.e. begin and end with one matched set of the seven PCR primer pairs. Many of the ROI (36%) did not match any primer pair on one or both ends (see Table 3). Using RNA-STAR, 74% of the ROI (including 94% of the full-length ROI) were successfully aligned uniquely to the rat genome. Of these aligned ROI, 99.5% aligned to the Ttc8 gene.

**Table 3:** Distribution of “reads of insert” (ROI) from the SMRT sequencing.

		# ROI	% ROI
Matches known, valid primer pair (“Full-Length”)		257,018	62.5%
Non Full length		153,963	37.5%
	No primer match	79,413	19.3%
	Matches known primer on one end	69,817	17.0%
Aligns uniquely to genome		305,675	74.4%
	Aligns to location *other than* Ttc8	1,550	0.4%
	Aligns to Ttc8 and is full-length	242,045	58.9%
Full-length and matches predicted isoform		180,479	43.9%
	Perfect full-length match to major isoform	56,977	13.9%
	Alignment match to major isoform	97,677	23.8%
	Matches major isoform with 8-9b intron retention	24,064	5.9%
	Matches isoform with minor splicing variant	1,761	0.4%
**Total Reads of Insert**		**410,981**	**100.0%**

**Fig 6:**
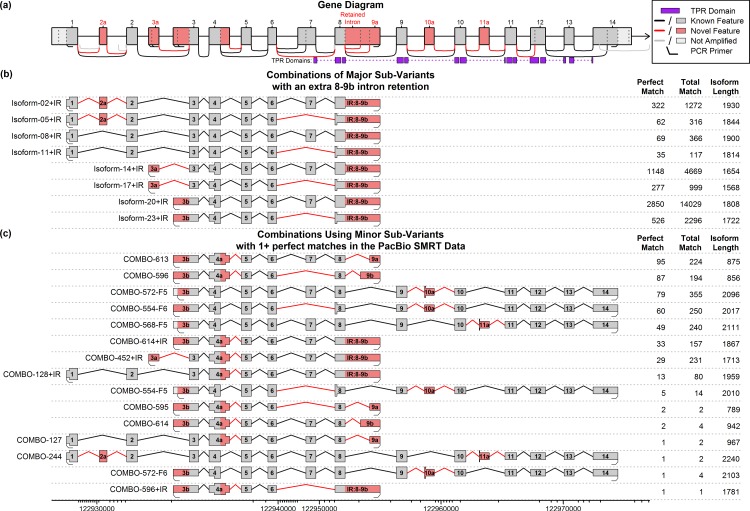
Additional novel isoforms discovered or validated via the PacBio SMRT sequence data. Part (a) displays the gene annotation, including the TPR domains (purple) and all alternative splice sites initially discovered by the Illumina RNA-Seq data. Part (b) displays all possible isoforms based on combinations of the major sub-variants plus an extra intron retention between exons 8 and 9b. Part (c) displays the subset of the 624 isoforms that involve one or more “minor” sub-variants (see Fig 3) that had one or more “perfect” matches in the PacBio SMRT data. Novel features are displayed in pink, and the widened region in each transcript denotes the largest open reading frame (ORF). The data tables on the right side of parts (b) and (c) display the number of full-length ROI from the SMRT sequencing that were found to match each isoform. The first column indicates perfect base-for-base, full-length matches, and the second column lists the total number of matches, including alignment matches in which the RNA-STAR aligner maps the ROI to the isoform. For a complete listing of all possible combinations of all known splice variations see the online supporting information. See S2 Dataset for more information on these and all other theoretical isoforms.

More than 50,000 ROI were found to be perfect, base-for-base, full-length matches to one of the 24 primary predicted combination isoforms (see Table 3, S4 Table). We used QoRTs [48] to identify near-perfect matches to predicted isoforms and found that an additional 97,677 ROI matched one of the 24 major sub-variant combinations (see Table 3, S4 Table, S3 Dataset). In addition, a large number of reads (24,064) were found to match one of the 24 primary combinations except with an additional intron retention between exons 8 and 9a (see Fig 6, S5 Table). An additional 1302 reads matched one of the broader set of 648 predicted combinations (see Fig 6, S6 Table, S2 Dataset).

Depending on the cutoff thresholds used, between 13 and 42 distinct isoforms were detected in the rat pineal gland. Thirteen distinct isoforms had more than 1000 perfect, base-for-base, primer-to-primer matches in the SMRT sequencing, 17 isoforms had 500 perfect matches, 31 had 50 perfect matches, and 42 isoforms had 1 or more perfect match. Note that the SMRT sequencing was conducted on a pool of pineal glands collected at a single time point (ZT19.5); additional isoforms may be present in this tissue at other time points. Thus, even with extremely conservative cutoff thresholds (500 perfect matches) we still detect more distinct isoforms (17 isoforms) than found in the Ensembl transcript annotations for rat (2 isoforms), mouse (6 isoforms), or human (15 isoforms).

It should be noted that since these isoforms were all based on splice junctions already detected in short-read high-throughput sequencing data, the chances that these isoforms are produced by sequencer artifacts is low. Short-read high-throughput RNA-Seq relies on fundamentally different chemistry compared with SMRT sequencing, and one would not expect these two methods to replicate one anothers’ biases or artifacts.

### Characterization of Ttc8 isoforms

The Ttc8 gene is part of a larger complex that serves to transport proteins in and out of cilia in certain cells [43], such as the outer segments of rod and cone photoreceptor cells [49]. The primary known functional domain of the Ttc8 gene consists of eight tetratricopeptide repeat (TPR) domains located in the C-terminal half of the protein (see Fig 4 and Fig 6). These repeats form a series of alpha-helices that together function as a protein-binding domain related to pilF [42].

Near the 5’ end of the “standard” transcript, exon 2a is a 30-base-pair-long cassette exon that preserves the open reading frame (ORF). The function of these additional 10 amino acids is unknown, but they only seem to be expressed in the pineal gland and retina, which (at least evolutionarily) feature prominent cilia [50, 51]. At least one isoform containing this cassette exon (isoform-03) has been shown in the existing literature to display substantial protein translation in mouse photoreceptor cells [39].

Further downstream, the two novel start sites in exon 3a and 3b both lead to the same alternative start codon in exon 3. This codon is in a Kozak consensus sequence and is in-frame with the standard Ttc8 transcript. Thus, major sub-variant combinations that combine the known 3’ end with the alternative exons 3a or 3b (isoforms 15 and 21, see Fig 4) have open reading frames containing all eight TPR domains, though they are missing the first 124 amino acids from the N-terminal end. There are two novel alternative 3’ terminal exons (9a and 9b) that have much shorter open reading frames than the known transcripts, and are missing at least 7 of the 8 TPR domains. The novel splice junction that causes exon 7 to be omitted results in a frame shift that introduces a stop codon near the 5’ end of the following exon (exon 8), excluding or truncating all 8 TPR domains. This means that several distinct transcripts that exclude exon 7 all contain identical ORFs, as the stop codon occurs upstream of the point of divergence.

There are also several other novel exons and splice junctions that were detected only at very low levels in the Illumina and PacBio datasets (see Fig 6). An elongation of exon 4 (“exon 4a”) introduces a novel stop codon and thus has a truncated ORF. The novel cassette exons 10a and 11a both introduce similar stop codons, producing ORFs that cover only 2 out of 8 and 3 out of 8 TPR domains, respectively.

Finally, the intron retention between exons 8 and 9b includes a stop codon located 5 amino acids into the intron. It is unclear whether the retained intron reads are actually representative of mature transcripts or if they were derived from immature mRNAs that were still in the process of being spliced. The PCR amplification performed prior to library preparation included a series of PCR reactions performed on size-selected fractions. Thus, even a small quantity of these (much larger) mRNAs could produce a disproportionate number of reads.

## Discussion

The results of this study provide interesting insights into the complex isoform population of the Ttc8 gene in the pineal gland and retina, and demonstrate the advantages of our hybrid, multi-platform sequencing strategy. These two points will be discussed sequentially in detail below.

### Differential start site usage

The evidence of differential start site usage of the Ttc8 gene in the rat pineal gland is comprehensive, consistent, and robust. We have detected and validated start-site-specific night-time upregulation of two novel transcription start sites belonging to this gene. This upregulation appears to be neurally-controlled by the SCG via NE and cAMP, as evidenced by the fact that *in vitro* treatment with NE and DBcAMP replicates this effect, and the fact that the effect is abrogated by removal or decentralization of the SCG. Furthermore, the existence of the novel start sites was validated using the PacBio SMRT sequencing platform (along with the isoforms to which they belong).

The existence and differential regulation of these novel start sites have been consistently confirmed in several different independent analyses using multiple sequencing/assay methods and experimental conditions. This comprehensive, multi-platform approach ensures that our results are replicable and robust, and greatly reduces the possibility that these observed phenomena could be the product of artifacts or biases.

### The Ttc8 gene

Although the exact function of this gene is not well understood, mutations in the gene have been implicated in non-syndromic retinitis pigmentosa (RP) [39, 40] and in Bardet-Biedl syndrome (BBS, which commonly includes RP among its symptoms) [41, 42]. At least one of the retina disease variants have been found in previous studies to be specifically related to the splicing of the gene’s transcripts. The cassette exon 2a is apparently expressed almost exclusively in the outer nuclear layer of the retina, and was absent from the ganglion cell layer, inner nuclear layer, and retinal pigment epithelium layers of the retina [40]. Similarly, this alternative exon was not found to be expressed in the brain, heart, kidney, liver, or testes. Despite the rarity of this splicing variant, previous studies have found that a specific point mutation just upstream of the 5’ acceptor site of this exon causes mis-splicing of the exon and results in nonsyndromic retinitis pigmentosa (RP) in humans [39, 40]. This particular mutation results in a frame-shift and premature stop codon in the 2a-inclusion isoforms, producing a non-functional protein. Other non-retinal tissues seem to be unaffected because they do not express exon 2a, resulting in RP rather than full BBS.

We found strong evidence that exon 2a is expressed in the pineal gland. In the discovery RNA-Seq dataset we find that around 2–3 times as many reads cover the splice junctions that include exon 2a (junctions N035 and N037, see Fig 1) compared with the junction that skips exon 2a (junction J019). Since reads are known to be non-uniform in their distribution these quantities may not be proportional to the actual expression levels, but the presence of these splice junctions in substantial quantities does at least indicate that the exon is expressed. The presence of this retina-specific exon is not totally unexpected given the phylogenetic and developmental relationship between the pineal gland and the photoreceptor cells in the retina. Our data suggests pedigrees affected by the nonsyndromic RP alleles may potentially have additional (undiagnosed) pleiotropic effects in the pineal gland, such as circadian timing issues related to reduced or impaired melatonin production. The two novel start site variants (exons 3a and 3b) contain a start codon that is in-frame with the known protein and lies upstream of the protein’s TPR domains. Thus, the hypothetical proteins produced by these isoforms would still include the full set of TPR domains and might thus still be functional.

We have also found numerous isoforms that use other downstream alternative splice junctions. Many of these isoforms have open reading frames that are missing most or all of these TPR domains, and thus could not possibly code for proteins that perform the same function as the standard Ttc8 isoform. It is possible that these isoforms code for proteins that use other (hitherto unknown) functional domains. Alternatively, these isoforms may actually represent long non-coding RNA’s, which have been found to have a substantial regulatory role in the rat endocrine system (and specifically in the rat pineal gland) [52, 53].

### Combinatorial isoforms

A set of 31 distinct novel isoforms (and 1 known isoform) was found that had 50 or more perfect, base-for-base matches in the PacBio data and that included only the splice junctions and exonic regions already detected in the Illumina dataset.

This is more than twice the number of isoforms found in human annotation (15 isoforms), and more than ten times the number found in the rat annotation (two isoforms). Furthermore, this was only a limited search: we only studied a single tissue, and only amplified three initial exons and two terminal exons. In contrast, the human annotation lists three known initial exons and five known terminal exons (Ensembl annotation, release 75).

In fact, we observe perfect full-length ROI that match most of the possible combinations of major exons and splice-junctions found in the short-read data (see Fig 4). However, there are some potential transcripts that have zero perfect matches, such as isoform 6 and isoform 12 (see Fig 4). These two isoforms would combine exon 1 with exon 14 and the novel skip of exon 7. All of these components are observed in other isoforms, many of which have high read coverage: exon 1 appears in isoforms 1 through 12, the novel skip of exon 7 appears in isoforms 4–6, 10–12, 16–18, and 22–24, and exon 14 appears in isoforms 3, 6, 9, 12, 15, 18, 21, and 24. However, the particular combination of exon 1, skipping exon 7, and then the set of exons 9–14, appears to never occur in detectable quantities. This is unlikely to be due to differential PCR amplification, since isoform 6 uses the same primers as isoform 3 (which had many ROI matches), and the two 2kb isoforms only differ by the presence or absence of the 86bp exon 7 in the middle. Furthermore, we observe numerous reads both with and without exon 7 that begin with exon 3a or 3b and end with exon 14 (see isoforms 15, 18, 21, and 24), and we observe numerous reads both with and without exon 7 that start with exon 1 but end with one of the novel end sites 9a or 9b.

The fact that there are some specific combinations of exons and splice junctions that are completely absent suggests that there are splicing regulatory mechanisms that act to correlate or “link” specific distant variable regions. For example: there could be some mechanism preventing the “full” isoforms (i.e. the isoforms that use exons 1 through 14) from splicing out exon 7, or there could be regulatory mechanisms that specifically splice out exon 7 some percentage of the time for the transcripts that use the alternative start sites 3a or 3b and/or alternative end sites 9a or 9b.

### Implications and relevance to future research

Although we have not established the function of the novel isoforms we have discovered (if any such function exists), this study has broad implications in the study of RNA expression and splicing. Even if these isoforms are not functional, knowledge of these isoforms is critical to the accuracy and validity of isoform-level abundance estimation methods. Such methods implicitly depend on the correctness and completeness of the isoform set (derived from an *a priori* transcript annotation and/or assembled from the data); the presence of a large number of unknown and/or undetectable isoforms will severely impact the accuracy of the estimates of the known isoforms.

Like several other studies that applied targeted, long-read sequencing of individual genes [36, 54], we found a very large number of novel isoforms. This strongly suggests that previous transcript discovery methods (predominantly based on high-throughput short-read RNA-Seq) were overly conservative and missed a large proportion of the true transcript population. This is consistent with the failure of the CuffLinks transcript assembly tool to detect any of the novel isoforms in our short-read RNA-Seq dataset (see S8 Fig, S1 Dataset). Long-read sequencing technologies offer a vast improvement over such methods, and as these technologies develop and become more widespread we may see a rapid increase in the number of known isoforms.

Furthermore, our results strongly suggest that numerous other genes also display differential transcript usage in the rat pineal gland between night and day, controlled by neural stimulation via the SCG. We found dozens of genes that displayed significant differential usage in all four “neurally-stimulated” experiments, whereas very few genes were detected in the SCGX and DCN experiments. Several of the neurally-regulated differentially-used genes were already well-known and extensively validated in the literature, including Crem [23–25], Pde4b [26], and Atp7b [28].

This differential transcript usage may be driven by the strong neurally-controlled night-time upregulation of the splicing regulator Mbnl2 [5, 14, 16]. This gene has been shown to regulate widespread but not universal alternative splicing in a wide range of species [18–22]. In fact, depletion of specific splicing factors, including Mbnl2, is thought to be a major cause of myotonic dystrophy because it results in dysregulation of splicing in specific transcripts [55]. It is possible that the daily 6-fold cycle of Mbnl2 in the pineal gland could regulate alternative splicing of many specific transcripts, including Ttc8.

### The value of the multi-platform approach

In this report data from three major technologies were used: strand-specific Illumina RNA-Seq, qPCR, and PacBio SMRT sequencing. None of these methods could, taken individually, can fully explain the regulatory processes that are taking place. The Illumina next-generation RNA-Seq dataset provided a transcriptome-wide survey of differential expression and differential splicing; however, the short reads limited our ability to identify full-length isoforms, and the various potential artifacts and biases made further validation necessary. qPCR was used to confirm the differential usage of specific targeted exons and splice junctions, and PacBio SMRT sequencing was used to identify the complete full-length isoforms. Only by combining the results from all three platforms were we able to develop a complete and comprehensive understanding of the regulatory phenomena that influence the expression of Ttc8 in the rat pineal gland.

## Methods

A more detailed descriptions of the precise methods used is available in the supplemental methods (see S1 Appendix). Animal use and care protocols were approved by the NIH Institutional Animal Care and Use Committee and followed the guidelines of the National Research Council's Guide for Care and Use of Laboratory Animals (Vol. 8) [56] and the Animal Research: Reporting In vivo Experiments (ARRIVE) guidelines [57].

### RNA-Seq Analyses

#### Discovery dataset

The surgical techniques, gland culture, sample collection, and sequencing is described in detail in a previous publication [14]. Briefly: the *in vivo* dataset consisted of four surgical groups: no surgical intervention (Control); neonatal sham surgery (Sham); neonatal bilateral SCG decentralization (DCN); neonatal bilateral superior cervical ganglionectomy (SCGX) [58]. Each surgical group was further subdivided into two sets and euthanized at either mid-day or midnight, generating a total of 8 biological conditions. The *in vitro* dataset consisted of 3 biological conditions: untreated control (untreated), norepinephrine-treated (NE), and dibutyryl-cyclic-AMP-treated (DBcAMP). For each of the 11 biological conditions, pineal glands were grouped into 3 biological replicates producing 33 biological replicates in total.

All rat RNA-Seq data were aligned to the rn6 rat genome build using the RNA-STAR aligner (v2.4.0j) [59], with the Ensembl transcript annotation (release 80) [60]. Quality control was carried out with the QoRTs quality control tool and no quality issues were detected [48]. Read counts for genes, exons, and splice junctions were also generated via QoRTs. A complete description of the sequencing and data processing can be found online (see section 1.1 of S1 Appendix). This dataset is publicly available in the NCBI gene expression omnibus (GEO), accession number GSE63309.

#### Analysis with JunctionSeq

The detection of differential usage of exons and splice junctions was performed using the JunctionSeq Bioconductor package [38]. The statistical methods used by this tool are fully described in the JunctionSeq methodology paper. Briefly, this package applies statistical methods similar to those used in the DEXSeq Bioconductor package, with modifications to allow for the detection of differential usage of known and novel splice junction loci [61, 62]. JunctionSeq, like DEXSeq, applies a multivariate generalized linear model using a negative binomial distribution to detect exons or splice junctions that are disproportionately used relative to the expression of their respective genes [38, 61]. Because it can query both known and novel features, this method provides superior performance over similar methods when using flawed or incomplete transcript annotations [38]. Most alternatives such as Kallisto [29], eXpress [30], and RSEM [31] cannot assess differential splicing unless the full-length isoforms are known.

The four *in vivo* analyses compared day and night conditions within each of the four surgical groups. The two *in vitro* analyses compared treated samples versus the untreated controls for the NE treatment and the DBcAMP treatment, respectively. Thus, each JunctionSeq analysis had two biological conditions (either night/day or treated/untreated) with three biological replicates per condition.

#### Time-series dataset

The time series RNA-Seq dataset is publicly available online from the National Center for Biotechnology Information (NCBI) Gene Expression Omnibus (GEO), accession number GSE46069. The sample collection, extraction, and sequencing methods for this previously-published [46] dataset are described in full on the GEO project page. Briefly: rat pineal and retinal tissue samples were taken at six time-points: ZT1, 7, 13, 15, 19, and 23. Only one sample was sequenced for each time-point. Alignment and data processing was performed using the same pipeline used for the discovery dataset.

### qPCR validation of differential transcription start site usage

qPCR was used to validate the day vs night difference in initial exon usage and whether this was affected by SCGX or DCN. Exon 2 was used as a proxy for usage of exon 1, since RNA-Seq showed that exon 2 is always included when exon 1 is used as the initial exon. A common reverse primer was used for all three alternative 5’ sub-variants: exon 2, exon 3a or exon 3b, each paired with a primer that matched exon 3 (see S1 Table). A more detailed description of the qPCR methods is available online (see section 1.3 of S1 Appendix).

### Identification of full-length novel isoforms

#### Sample collection and preparation

To obtain representative samples of all the transcripts present in the pineal gland, a cDNA library constructed from rat pineal glands collected at ZT19.5 were amplified using seven different pairs of primers covering combinations of the three alternative initial exons and the two alternative terminal exons (S2 Table). The products of the seven PCR runs were pooled, prepared, and run on a Pacific Biosciences (PacBio) RS II sequencer in seven SMRT cells. “Sub-reads” were compiled into consensus “reads-of-insert” (ROI) using the SMRT Pipe software suite provided by Pac Bio. The PacBio SMRT sequencing data has been deposited online in the NCBI short read archive (SRA), accession ID SRX1736990. Further details on the PacBio SMRT sequencing methods are available online (see section 1.4.1–1.4.2 in S1 Appendix).

#### Analysis

We did not use the “Quiver” methodology proposed by PacBio to perform our transcript assembly, as this method was designed for use with different experimental designs and performed poorly under our conditions. Specifically: Quiver appears to have been designed for a different type of PCR amplification in which the primers were not expected to match the genomic template (and thus contigs are not required to begin and end with primer sequence), and where the “full-length” ROI were not required to begin and end with the start/end sites. This meant that in many cases it produced assemblies and read assignments that were obviously incorrect. For example: the major theoretical combination 15 (see Fig 4) was not detected, and the (numerous) reads that perfectly matched this isoform were instead matched to a partial segment of a larger isoform that began with exon 1. Our experimental design actually made the assembly much simpler. Our long-read sequence data had relatively low diversity (see S10 Fig), and we already had an *a priori* set of theoretical isoforms derived from the short read RNA-Seq data. Thus, in order to confirm the existence of these isoforms we did not need the advanced isoform assembly implemented by Quiver.

The ROI were matched to primer pairs using Phmmer [63], using the exact same method implemented by Quiver. However, unlike with Quiver, the primer sequences were not clipped off. The ROI were then aligned to the rat genome (build rn6) using the RNA-STARlong aligner (v2.4.2a), which was specifically designed for use on long reads of this type. These aligned ROI were then compared to the theoretical isoform set using the new “longReadClassifier” function of the QoRTs software package [48]. This tool counts the number of ROI that match by alignment to each isoform. An ROI was considered a “perfect match” if it was an exact, base-for-base, primer-to-primer match to the theoretical isoform. An ROI was considered an “alignment match” if and only if they covered all the isoform’s exons, spliced across all the isoform’s splice junctions, covered no other exons or splice junctions, and if both of the alignment ends were within 3 base pairs of the primer endpoints. Further details on the PacBio SMRT sequencing analyses are available online (see section 1.4.3–1.4.4 in S1 Appendix).

## Supporting Information

S1 Appendix Supplemental methods and additional information. (DOCX)

S1 Dataset (gtf.gz) A transcript assembly GTF file generated by CuffLinks/CuffMerge (v2.0.2). These transcripts were generated via a CuffLinks run on each sample in the discovery set, followed by the use of CuffMerge to merge these individual-sample assemblies. Note that CuffLinks failed to discover any of the novel transcripts, and there are no transcripts covering any of the 17 novel splice junctions observed in the Illumina and PacBio datasets.
(GZ) Click here for additional data file.

S2 Dataset (txt) A tab-delimited text file that describes all theoretical isoforms. It includes columns listing the ROI counts from the PacBio SMRT sequencing.
(TXT)

S3 Dataset (txt) A tab-delimited text file that describes each of the “major” sub-variant combinations. It includes columns listing the ROI counts from the PacBio SMRT sequencing. See also Fig 4C.
(TXT)

S4 Dataset (xlsx) JunctionSeq results. An excel file containing the JunctionSeq results for each analysis, for all genes that showed significant differential usage.
(XLSX)

S5 Dataset (gff.gz) A gzip-compressed GFF file containing the JunctionSeq annotation. This includes all exonic regions and known/novel splice junctions and their unique identifiers.
(GZ) Click here for additional data file.

S6 Dataset (xlsx) An excel spreadsheet with the 42 novel isoforms. This includes all theoretical isoforms that had one or more perfect, full-length matches in the PacBio dataset. Also included is the GenBank accession numbers for each isoform.
(XLSX)

S1 Fig Venn diagram of the four JunctionSeq analyses genes (p-adjust < 0.0001). This Venn diagram displays the overlap between the genes detected as containing a differentially used feature in each of the four JunctionSeq “stimulus” analyses at the adjusted-p-value < 0.0001 level.
(PNG)

S2 Fig JunctionSeq results for the Ttc8 gene, control night/day experiment. (PNG)

S3 Fig JunctionSeq results for the Ttc8 gene, Untreated vs NE experiment. (PNG)

S4 Fig JunctionSeq results for the Ttc8 gene, Untreated vs DBcAMP experiment. (PNG)

S5 Fig JunctionSeq results for the Ttc8 gene, DCN (Dec) night/day experiment. (PNG)

S6 Fig JunctionSeq results for the Ttc8 gene, SCGX (Ex) night/day experiment. (PNG)

S7 Fig RNA-Seq data from the rat pineal gland sampled at various times during the day. Rats were housed in a 14:10 light:dark cycle. ZT, Zeitgeber time.
(PNG)

S8 Fig CuffLinks assembly for the region shown in Fig 2. As you can see, there are 30–60 read pairs per sample covering junction N040 in the innervated sample groups (Ctrl_N, Sham_N, DBcAMP and NE). However, CuffLinks does not detect any transcripts containing this junction. It also fails to detect novel start site 3b despite substantial read coverage over this region. Subsequent validation proved that these novel splice sites are real.
(PNG)

S9 Fig Images of the PCR products of reactions that were pooled for SMRT sequencing. The label above each lane indicates the reaction number and the primer pair that was used for amplification. See S2 Table for primer sequences.
(PNG)

S10 Fig Read lengths from PacBio SMRT sequencing. This plot shows a base-pair-resolution histogram of the read-of-insert (ROI) length produced by the PacBio SMRT sequencing of the Ttc8 gene. The reads are separated by primer pair and drawn separately in each color. The lengths of major potential predicted isoforms are marked at the bottom, along with the potential isoform ID. Reads of insert that did not match a legal primer pair at both ends were assumed to be non-full-length (non-FL) and are plotted in gray. Note that due to the accuracy of the SMRT sequencing platform, the presence of many of the predicted isoforms can be easily recognized based only on the high number of ROI at that exact length. Also note that this effect is less pronounced for the longer isoforms, due to the fact that the ROI accuracy is inversely associated with template length.
(PNG)

S11 Fig The same information displayed in S10 Fig, separated by primer pair matching. (PNG)

S12 Fig RNA-Seq data from the rat retina sampled at various times during the day. Rats were housed in a 14:10 light:dark cycle. Note that there are 2 samples at ZT7. Note that there are no reads covering the novel exon 3a, nor covering the region specific to novel exon 3b. ZT, Zeitgeber time.
(PNG)

S13 Fig Various transcript annotations and gene predictions for the Ttc8 gene. The above figure displays the gene annotations and gene predictions from Ensembl, RefSeq, Augustus, the mammalian gene collection (MGC), SGP, and geneid. Note that none of the novel exons or splice junctions appear in any of the transcript annotations. Note that the Rat Genome Database (RGD) is not included in this plot because it has not yet been lifted over to the rn6 rat genome build. See (S15 Fig) for the RGD annotation.
(PNG)

S14 Fig Detailed examination of the various transcript annotation databases on the exon 2–5 region of the Ttc8 gene. This figure is identical to the previous, except zoomed in on the region containing the major novel exons 3a and 3b. Note that none of the novel exons or splice junctions appear in any of the transcript annotations.
(PNG)

S15 Fig Transcript annotations from various databases on the older rn4 rat genome assembly. Note that this assembly includes the RGD curated gene database. Additionally, note the presence of transcript ENSRNOT00000006199 in the ensembl annotation (release 69), which was absent in subsequent ensembl releases. This transcript includes one of the “novel” exons detected in our analyses, exon 2a. The transcript appears to have been lost in the liftover to rn5 (possibly due to a nearby gap in the rn5 genome build), and was never re-added in subsequent releases. Note that none of the other novel exons or splice junctions appear in any of the transcript annotations.
(PNG)

S1 Table qPCR primer pairs used in the quantitation of start site usage. These primers were used to confirm and quantitate the differential expression of the three start sites of the Ttc8 gene. The first primer pair addresses the annotated Ttc8 start site, and primer pairs 2 and 3 address the two major novel start sites discovered in the Illumina RNA-Seq data.
(DOCX)

S2 Table PCR primer pairs used for PacBio SMRT sequencing. These primers were used to amplify the transcripts of the Ttc8 gene prior to sequencing with the PacBio SMRT platform. Note that primers F3 and F4 differ by only 4 bases, and primers F5 and F6 differ by only 6.
(DOCX)

S3 Table Additional size-selected amplification for PacBio Sequencing. In some cases additional size selection and amplification was necessary, as per the PacBio SMRT protocol. This table lists the size windows, primers, cycle counts, and total yield.
(DOCX)

S4 Table Read-of-Insert counts for the 24 “major sub-variant” potential isoforms. These are the counts for ROI that match one of the 24 potential major isoforms (see Fig 3 and Fig 4).
(DOCX)

S5 Table Read-of-Insert counts for predicted potential isoforms with an added intron retention between exons 8 and 9b. See also Fig 6B.
(DOCX)

S6 Table Read-of-Insert counts for various extremely-low-coverage sub-variants. See also **Fig 3**. Note that these counts are the number of ROI that perfectly match any of the theoretical potential isoforms that includes the given sub-variant. For example the first row (late donor, exon 4) compiles the counts from 8 different isoforms, all of which contain the late donor alternative splice junction on exon 4, as seen in **Fig 6C**.
(DOCX)
