# Supplementary material for: Alternative Isoform Analysis of Ttc8 Expression in the Rat Pineal Gland Using a Multi-Platform Sequencing Approach Reveals Neural Regulation
Source: PLoS One. 2016 Sep 29;11(9):e0163590. doi: 10.1371/journal.pone.0163590 (PMC5042479; doi:10.1371/journal.pone.0163590)
Supplement: S1 Appendix — (DOCX) [file pone.0163590.s001.docx]

Appendix S1

Alternative isoform analysis of Ttc8 expression in the rat pineal gland using a multi-platform sequencing approach reveals neural regulation

Stephen W. Hartley, James C. Mullikin, David C. Klein, Morgan Park, NISC Comparative Sequencing Program, Steven L. Coon

Table of Contents

[Supplemental Methods 2](#_Toc451156957)

[Discovery dataset (Illumina RNA-Seq) 2](#_Toc451156958)

[Rationale for selecting Ttc8 for validation 2](#_Toc451156959)

[qPCR quantitation of differential transcription start site usage 3](#_Toc451156960)

[RNA extraction and cDNA synthesis. 3](#_Toc451156961)

[Quantitative real-time PCR (qPCR) 3](#_Toc451156962)

[Identification of full-length novel isoforms 3](#_Toc451156963)

[Sample collection and preparation 3](#_Toc451156964)

[Library preparation and Sequencing 4](#_Toc451156965)

[Preliminary Quiver analysis 4](#_Toc451156966)

[Analysis via RNA-STAR and QoRTs 5](#_Toc451156967)

[NISC Comparative Sequencing Program Authors 5](#_Toc451156968)

[Browser Tracks 6](#_Toc451156969)

[Supplemental Tables and Figures 6](#_Toc451156970)

[Supplemental Tables 6](#_Toc451156971)

[Supplemental Figures 10](#_Toc451156972)

[List of Supplemental Datasets 22](#_Toc451156973)

[References 23](#_Toc451156974)

[List of Main Text Tables and Figures 24](#_Toc451156975)

# Supplemental Methods

## Discovery dataset (Illumina RNA-Seq)

All rat RNA-Seq data were aligned to the rn6 rat genome build using the RNA-STAR aligner (v2.4.0j) [1], with the Ensembl transcript annotation (release 80) [2]. Quality control was carried out with the QoRTs quality control tool, and no quality issues were detected [3]. Read counts for genes, exons, and splice junctions were also generated via QoRTs. Read counts were restricted to protein coding and lincRNA transcripts. All genes that overlapped with one or more other genes were excluded from all analyses. Our objective in this analysis was to identify alternative isoform regulation rather than differential gene expression, and when genes overlap it can be very difficult to distinguish the two phenomena. This excluded 229 genes from the analyses.

Novel splice junctions were added to the dataset if and only if they could be uniquely assigned to a known gene and had a mean normalized coverage of at least 1 read-pair per sample. This added a total of 28,170 novel splice junctions across 8,090 genes. Additionally, 29,881 novel splice junctions passed the coverage threshold but did not belong to any known gene, and 1,246 novel splice junctions passed this threshold but spliced between the intergenic regions of two or more non-overlapping genes. Only the junctions that could be uniquely assigned to a single gene were included in the analyses.

Animal use and care protocols were approved by the NIH Institutional Animal Care and Use Committee and followed the guidelines of the National Research Council's Guide for Care and Use of Laboratory Animals (Vol. 8) [4] and the Animal Research: Reporting In vivo Experiments (ARRIVE) guidelines [5].

## Rationale for selecting Ttc8 for validation

No matter how robust the statistical methodology, any differential effects detected purely from short-read RNA-Seq data must be independently validated in order to be considered credible. However, such validation is expensive and time-consuming. Out of the hundreds of detected effects we decided to select only one for validation.

We thus restricted our candidate gene lists to genes with strong (FC > 2) and statistically significant (adjusted-p < 0.0001) differential usage. Since we wanted to demonstrate our ability to detect differentials in unannotated splice junctions, we prioritized genes that had novel splice junctions and showed differential usage of these junctions or of nearby known junctions and exons.

This left us with a handful of potential genes (see Table 2). We eliminated Crem [6-8], Pde4b [9], and Atp7b [10] from consideration because the differential isoform usage of these genes are already well-characterized in the literature.

The remaining candidates were further examined using the UCSC genome browser tracks generated by the QoRTs/JunctionSeq pipeline [3]. These tracks allowed us to check for a number of potential artifacts and biases that could produce false discoveries. For example: the UCSC browser default tracks include a RepeatMasker track that displays repetitive or low-complexity regions. Such regions could cause apparent differentials via mapping artifacts. Similarly, regions of cross-strand overlap between genes could cause false discoveries, as the strand-specific library prep protocol has variable efficacy. The UCSC browser also allowed us to create and test potential PCR primer pairs.

Candidate genes were qualitatively assessed based upon the strength and significance of the differential usage, the consistency of the gene expression profiles between analyses, whether the effect could possibly be caused by a mapping or library-prep artifact, the ease of which the gene could be validated, and potential biological relevance. Considering these criteria, we selected the gene Ttc8.

## qPCR quantitation of differential transcription start site usage

### RNA extraction and cDNA synthesis.

Total RNA was extracted using Trizol reagent (ThermoFisher, Waltham, MA) followed by clean-up and on-column DNase treatment using an RNeasy micro kit (Qiagen, Germantown, MD). cDNA was synthesized from 1 µg total RNA using the Superscript III kit (ThermoFisher) and 250 ng random hexamers (Roche, Indianapolis, IN). RNAs and cDNAs were produced from triplicate pools of pineal glands from each of the eight treatment groups: animals sacrificed at mid-day (ZT7) and mid-night (ZT19) from each of the four surgical groups (Control, Sham, SGCX and DCN). In addition, a separate cDNA was synthesized from pineal glands from rat sacrificed at ZT19.5.

### Quantitative real-time PCR (qPCR)

qPCR was used to validate the day vs night difference in initial exon usage and whether this was affected by SCGX or DCN. Exon 2 was used as a proxy for usage of exon 1, since RNA-Seq showed that exon 2 is always included when exon 1 is used as the initial exon. A common reverse primer was used for all three alternative starting exons: exon 2, exon 3a or exon 3b, each paired with a primer that matched exon 3 (see S1 table).

qPCRs were performed in a 20 µl reaction using RT2 SYBR Green qPCR MasterMix (Qiagen, Germantown, MD), in a LightCycler 480 Real-Time qPCR System (Roche, Indianapolis, IN), using the following conditions: 95oC for 2 min, then cycles of 95°C for 15 sec, 60°C for 30 sec and 72°C for 30 sec.

Data were normalized to Gapdh expression in each sample (primers in S1 table). Relative expression levels were calculated using separate standard curves for each primer pair and a dilution series of pooled cDNAs.

## Identification of full-length novel isoforms

### Sample collection and preparation

To obtain representative samples of all the transcripts present in the pineal gland, a cDNA library constructed from rat pineal glands collected at ZT19.5 were amplified using seven different pairs of primers covering combinations of the three alternative initial exons and the two alternative terminal exons (S2 table). Amplification was performed in a duplicate 25 µl reactions using Phusion Hot Start II polymerase (ThermoFisher, Waltham, MA) using the following conditions: 98ºC for 30 sec, then 35 cycles of 98ºC for 10 sec, 64ºC for 15 sec and 72ºC for 90 sec, then 72ºC for 5 min. The results were verified by running on a 1% agarose gel (see S9 figure). Equal volumes of each PCR reaction were pooled and purified using a NucleoSpin Gel and PCR Cleanup kit (Macherey-Nagel, Bethlehem, PA) before being used for library construction for PacBio sequencing.

The seven amplicons were pooled and size-selected on a 0.8% low melt agarose gel. Regions corresponding to 700-1200bp, 1200-2200bp, and ~3000bp were extracted using QIAQuick Gel Extraction Kit (Qiagen). Fractions 1200-2200 and ~3000 were re-amplified to increase the amount of sample using the Kapa HiFi Ready Mix 2x (Kapa Biosystems), with the primers and cycles indicated in S3 table.

### Library preparation and sequencing

Libraries were prepared using the SMRTbell Template Prep kit 1.0 (Pacific Biosciences) using the “2 kb Template Preparation and Sequencing” protocol. SMRTbell templates were bound to Polymerase P6 using the DNA/Polymerase Binding Kit P6 and primer from the template kit. Polymerase-template complexes were bound to MagBeads using the MagBead Binding Kit.

Sequencing was carried out using seven SMRT cells on a PacBio RS II sequencer using C4 sequencing reagents and a loading concentration of 0.003-0.01nM to generate 400k reads of insert (ROI) for fraction 1, 14k for fraction 2, and 45k reads for fraction 3. Movie lengths were 240 minutes for each SMRTcell.

Sub-reads were compiled into reads of insert (ROI) using the PacBio-provided software pipeline. This yielded a total of 410,981 reads-of-insert (ROI) that passed the (default) quality and consensus filters.

### Preliminary Quiver analysis

While the Quiver methodology has been shown to be very accurate under the conditions for which it was designed, out experimental design had a number of key differences that made the Quiver analysis pipeline poorly suited to our purposes.

During testing, we ran the Quiver pipeline on a subset of the data composed of the output of 4 SMRTcells. This yielded 220,721 reads of insert, with 135,165 ROI being classified as “full length”. Quiver identified 219 high-quality contigs, including 11 out of 24 of the “major” sub-variant combinations (missing combinations 05, 06, 11, 12, 15, 17, and 18; see Fig 4). Some of these isoforms were missed due to the fact that they have very little support in the data. However, a few of the isoforms were missed despite the fact that they had thousands of perfect base-for-base, primer-to-primer ROI supporting them: specifically combination 15 (see Fig 4) and 14-IR (see Fig 5), both of which included exon 3a. Upon further investigation we found that the reads that matched these isoforms were assigned by Quiver to partial fragments of two different larger isoforms (which did not include exon 3a). In addition, Quiver produced a large number of contigs that differed only by their endpoints, which generally did not match up with the primer loci. All true contigs would, in our experimental design, begin and end with the primer sequence, and thus we could discount these contigs as artifacts caused by the inapplicability of the Quiver methodology.

Based on these preliminary results, we elected not to proceed with Quiver for our final analysis.

### Analysis via RNA-STAR and QoRTs

The ROI were matched to primer pairs using Phmmer [11], the same method implemented by Quiver. However, unlike with the Quiver method the primer sequences were not clipped off. The ROI were then aligned to the rat genome (build rn6) using the RNA-STARlong aligner (v2.4.2a), which is specifically designed for long reads of this type. These aligned ROI were then compared to the theoretical isoform set using the new “longReadClassifier” function of the QoRTs software package [3]. This tool counts the number of ROI that match by alignment to a particular isoform. An ROI was considered a perfect match if it was an exact, base-for-base, primer-to-primer match to the theoretical isoform. An ROI was considered an “alignment match” if and only if it covered all the isoform’s exons, spliced across all the isoform’s splice junctions, covered no other exons or splice junctions, and if both of the alignment ends were within 3 base pairs of the primer endpoints.

# NISC Comparative Sequencing Program Authors

Betty Barnabas, PhD; Robert Blakesley, PhD; Gerry Bouffard, PhD; Shelise Brooks, BS; Holly Coleman, MSc; Mila Dekhtyar, MSc; Michael Gregory, MSc; Xiaobin Guan, PhD; Jyoti Gupta, MSc; Joel Han, BS; Shi-ling Ho, BS; Richelle Legaspi, MSc; Quino Maduro, BS; Cathy Masiello, MSc; Baishali Maskeri, PhD; Jenny McDowell, PhD; Casandra Montemayor, MSc; James Mullikin, PhD; Morgan Park, PhD; Nancy Riebow, BS; Karen Schandler, MSc; Brian Schmidt, BS; Christina Sison, BS; Mal Stantripop, BS; James Thomas, PhD; Pam Thomas, PhD; Meg Vemulapalli, MSc; Alice Young, BA.

# Browser Tracks

Publicly-available UCSC browser tracks, similar to the tracks used in Fig **2**, are available online displaying the JunctionSeq results (exon/junction expression levels and significant p-values) alongside “wiggle” plots displaying the mean normalized coverage for 20-bp windows across the genome.

- [Control group, day vs night (in vivo)](https://genome.ucsc.edu/cgi-bin/hgTracks?hgS_doOtherUser=submit&hgS_otherUserName=stephen.hartley&hgS_otherUserSessionName=rn6_PUBLIC_HUB_CTRL)
- [Sham group, day vs night (in vivo)](https://genome.ucsc.edu/cgi-bin/hgTracks?hgS_doOtherUser=submit&hgS_otherUserName=stephen.hartley&hgS_otherUserSessionName=rn6_PUBLIC_HUB_SHAM)
- [Untreated vs DBcAMP-treated (in vitro)](https://genome.ucsc.edu/cgi-bin/hgTracks?hgS_doOtherUser=submit&hgS_otherUserName=stephen.hartley&hgS_otherUserSessionName=rn6_PUBLIC_HUB_CNvDB)
- [Untreated vs NE-treated (in vitro)](https://genome.ucsc.edu/cgi-bin/hgTracks?hgS_doOtherUser=submit&hgS_otherUserName=stephen.hartley&hgS_otherUserSessionName=rn6_PUBLIC_HUB_CNvNE)
- [SCGX group, day vs night (in vivo)](https://genome.ucsc.edu/cgi-bin/hgTracks?hgS_doOtherUser=submit&hgS_otherUserName=stephen.hartley&hgS_otherUserSessionName=rn6_PUBLIC_HUB_SCGX)
- [DCN group, day vs night (in vivo)](https://genome.ucsc.edu/cgi-bin/hgTracks?hgS_doOtherUser=submit&hgS_otherUserName=stephen.hartley&hgS_otherUserSessionName=rn6_PUBLIC_HUB_DCN)

# Supplemental Tables and Figures

The supplemental tables and figures are available individually online, and are compiled here for convenience.

## Supplemental Tables

S1 table: qPCR primer pairs used in the quantitation of start site usage. These primers were used to confirm and quantitate the differential expression of the three start sites of the Ttc8 gene. The first primer pair addresses the annotated Ttc8 start site, and primer pairs 2 and 3 address the two major novel start sites discovered in the Illumina RNA-Seq data.

| **Reaction** | **Primer Pair** | | **Primer Sequence** | |
| --- | --- | --- | --- | --- |
|  | **Forward** | **Reverse** | **Forward** | **Reverse** |
| 1 | F14 (exon 2) | R7 (exon 3) | CGTGGACCAGGAAGGGATTG | GACAGCTTGAGTCGGTCCTC |
| 2 | F4 (exon 3a) | R7 (exon 3) | GATACGGCTTTGCTGGCGAT | GACAGCTTGAGTCGGTCCTC |
| 3 | F16 (exon 3b) | R7 (exon 3) | TCTTTCCCAGCCCAGTACAAG | GACAGCTTGAGTCGGTCCTC |
| 4 | Gapdh-F1 | Gapdh-R1 | TGGTGAAGGTCGGTGTGAACGGAT | TCCATGGTGGTGAAGACGCCAGTA |

S2 table: PCR primer pairs used for PacBio SMRT sequencing. These primers were used to amplify the transcripts of the Ttc8 gene prior to sequencing with the PacBio SMRT platform. Note that primers F3 and F4 differ by only 4 bases, and primers F5 and F6 differ by only 6.

| # | **Primer Pair** | | **Primer Sequence** | | **Genomic Position** | |
| --- | --- | --- | --- | --- | --- | --- |
|  | **Forward** | **Reverse** | **Forward** | **Reverse** | **Start** | **End** |
| 1 | F1 (exon1) | R1 (exon14 ) | CACAGGACCTTTGAGCTCGT | TCTGACCTAAGTTTTCAACGATAGC | 122920371 | 122974377 |
| 2 | F3 (exon3a) | R1 (exon14) | CGGCTTTGCTGGCGATTTAT | TCTGACCTAAGTTTTCAACGATAGC | 122936579 | 122974377 |
| 3 | F5 (exon3b) | R1 (exon14) | ATGCCACCAAACAGAGCAGT | TCTGACCTAAGTTTTCAACGATAGC | 122937064 | 122974377 |
| 4 | F6 (exon3b) | R1 (exon14) | CAGGTTCATGCCACCAAACAG | TCTGACCTAAGTTTTCAACGATAGC | 122937057 | 122974377 |
| 5 | F1 (exon1) | R8 (exon9a) | CACAGGACCTTTGAGCTCGT | TGTGCCTGGTAACCTTGGAG | 122920371 | 122954254 |
| 6 | F4 (exon3a) | R8 (exon9a) | GATACGGCTTTGCTGGCGAT | TGTGCCTGGTAACCTTGGAG | 122936575 | 122954254 |
| 7 | F6 (exon3b) | R8 (exon9a) | CAGGTTCATGCCACCAAACAG | TGTGCCTGGTAACCTTGGAG | 122937057 | 122954254 |

S3 table: Additional size-selected amplification for PacBio sequencing. In some cases additional size selection and amplification was necessary, as per the PacBio SMRT protocol. This table lists the size windows, primers, cycle counts, and total yield.

| **Fraction Number** | **Amplicon Size Range** | **PCR Primers** | **Number of Cycles** | **Total Yield** |
| --- | --- | --- | --- | --- |
| 1 | 600-1200 bp | None | 0 | 169 ng |
| 2 | 1200-2200 bp | F1/R1  F3/R1  F5/R1  F6/R1  F1/R8  F4/R8  F6/R8 | 6  6  6  6  6  6  6 | 600 ng |
| 3 | ~3000 bp | F1/R1  F6/R8 | 12  12 | 1584 ng |

S4 table: Read-of-Insert counts for the 24 “major sub-variant” potential isoforms. These are the counts for ROI that match one of the 24 potential major isoforms (see Fig 3 and Fig 4).

| **Isoform**  **ID** | **Primer**  **Pair** | **Length** | **# Perfect Match** | **# Align**  **Match** | **Total**  **Match** |
| --- | --- | --- | --- | --- | --- |
| 01 | F1-R8 | 938 | 10390 | 10328 | 20718 |
| 02 | F1-R8 | 1005 | 3695 | 3753 | 7448 |
| 03 | F1-R1 | 2151 | 1630 | 9785 | 11415 |
| 04 | F1-R8 | 852 | 202 | 184 | 386 |
| 05 | F1-R8 | 919 | 3 | 1 | 4 |
| 06 | F1-R1 | 2065 | 0 | 1 | 1 |
| 07 | F1-R8 | 908 | 1422 | 2100 | 3522 |
| 08 | F1-R8 | 975 | 917 | 1206 | 2123 |
| 09 | F1-R1 | 2121 | 153 | 1490 | 1643 |
| 10 | F1-R8 | 822 | 111 | 128 | 239 |
| 11 | F1-R8 | 889 | 70 | 57 | 127 |
| 12 | F1-R1 | 2035 | 0 | 0 | 0 |
| 13 | F4-R8 | 662 | 10601 | 11764 | 22365 |
| 14 | F4-R8 | 729 | 2798 | 3444 | 6242 |
| 15 | F3-R1 | 1871 | 2222 | 10599 | 12821 |
| 16 | F4-R8 | 576 | 170 | 186 | 356 |
| 17 | F4-R8 | 643 | 27 | 35 | 62 |
| 18 | F3-R1 | 1785 | 194 | 480 | 674 |
| 19 | F6-R8 | 816 | 13600 | 16684 | 30284 |
| 20 | F6-R8 | 883 | 2947 | 3495 | 6442 |
| 21 (F6) | F6-R1 | 2029 | 1571 | 8491 | 10062 |
| 21 (F5) | F5-R1 | 2022 | 1323 | 7397 | 8720 |
| 22 | F6-R8 | 730 | 1400 | 1697 | 3097 |
| 23 | F6-R8 | 797 | 801 | 905 | 1706 |
| 24 (F6) | F6-R1 | 1943 | 483 | 2347 | 2830 |
| 24 (F5) | F5-R1 | 1936 | 247 | 1120 | 1367 |
| **TOTAL:** | | | **56977** | **97677** | **154654** |

S5 table: Read-of-Insert counts for predicted potential isoforms with an added intron retention between exons 8 and 9b. See also Fig 6b.

| **Isoform**  **ID** | **Primer**  **Pair** | **Length** | **# Perfect Match** | **# Align**  **Match** | **Total**  **Match** |
| --- | --- | --- | --- | --- | --- |
| 02-IR | F1-R8 | 1930 | 322 | 950 | 1272 |
| 05-IR | F1-R8 | 1844 | 62 | 254 | 316 |
| 08-IR | F1-R8 | 1900 | 69 | 297 | 366 |
| 11-IR | F1-R8 | 1814 | 35 | 82 | 117 |
| 14-IR | F4-R8 | 1654 | 1148 | 3521 | 4669 |
| 17-IR | F4-R8 | 1568 | 277 | 722 | 999 |
| 20-IR | F6-R8 | 1808 | 2850 | 11179 | 14029 |
| 23-IR | F6-R8 | 1722 | 526 | 1770 | 2296 |
| TOTAL: | | | 5289 | 18775 | 24064 |

S6 table: Read-of-Insert counts for various extremely-low-coverage sub-variants. See also Fig 3. Note that these counts are the number of ROI that perfectly match any of the theoretical potential isoforms that includes the given sub-variant. For example the first row (late donor, exon 4) compiles the counts from 8 different isoforms, all of which contain the late donor alternative splice junction on exon 4, as seen in Fig 5c.

| **Sub-variant**  **Description** | **# Belonging to Perfect Match** | **# Belonging to Align**  **Match** | **Total Belonging to Match** |
| --- | --- | --- | --- |
| Late donor, exon 4 (exon 4a) | 263 | 632 | 895 |
| Skip exon 5 | 0 | 0 | 0 |
| Include 7a | 0 | 0 | 0 |
| Extra cassette exon 10a | 145 | 478 | 623 |
| Extra cassette exon 11a | 50 | 192 | 242 |
| Early acceptor, exon 12 (exon 12a) | 0 | 0 | 0 |
| Skip exon 13, end with exon 14a | 0 | 1 | 1 |
| **Totals:** | **458** | **1303** | **1761** |

## Supplemental Figures


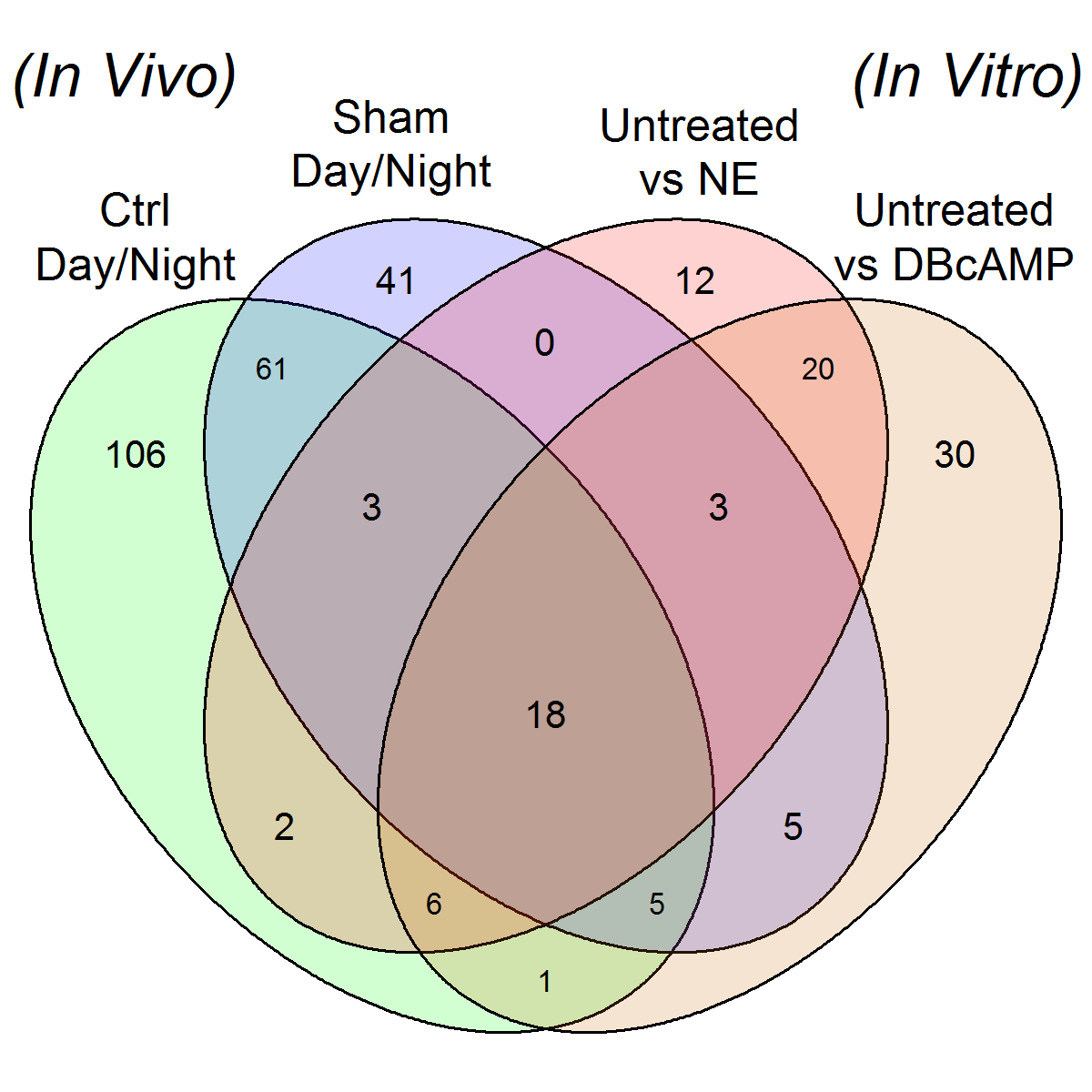


S1 figure: Venn diagram of the four JunctionSeq analyses genes (p-adjust < 0.0001). This Venn diagram displays the overlap between the genes detected as containing a differentially used feature in each of the four JunctionSeq “stimulus” analyses at the adjusted-p-value < 0.0001 level.

**
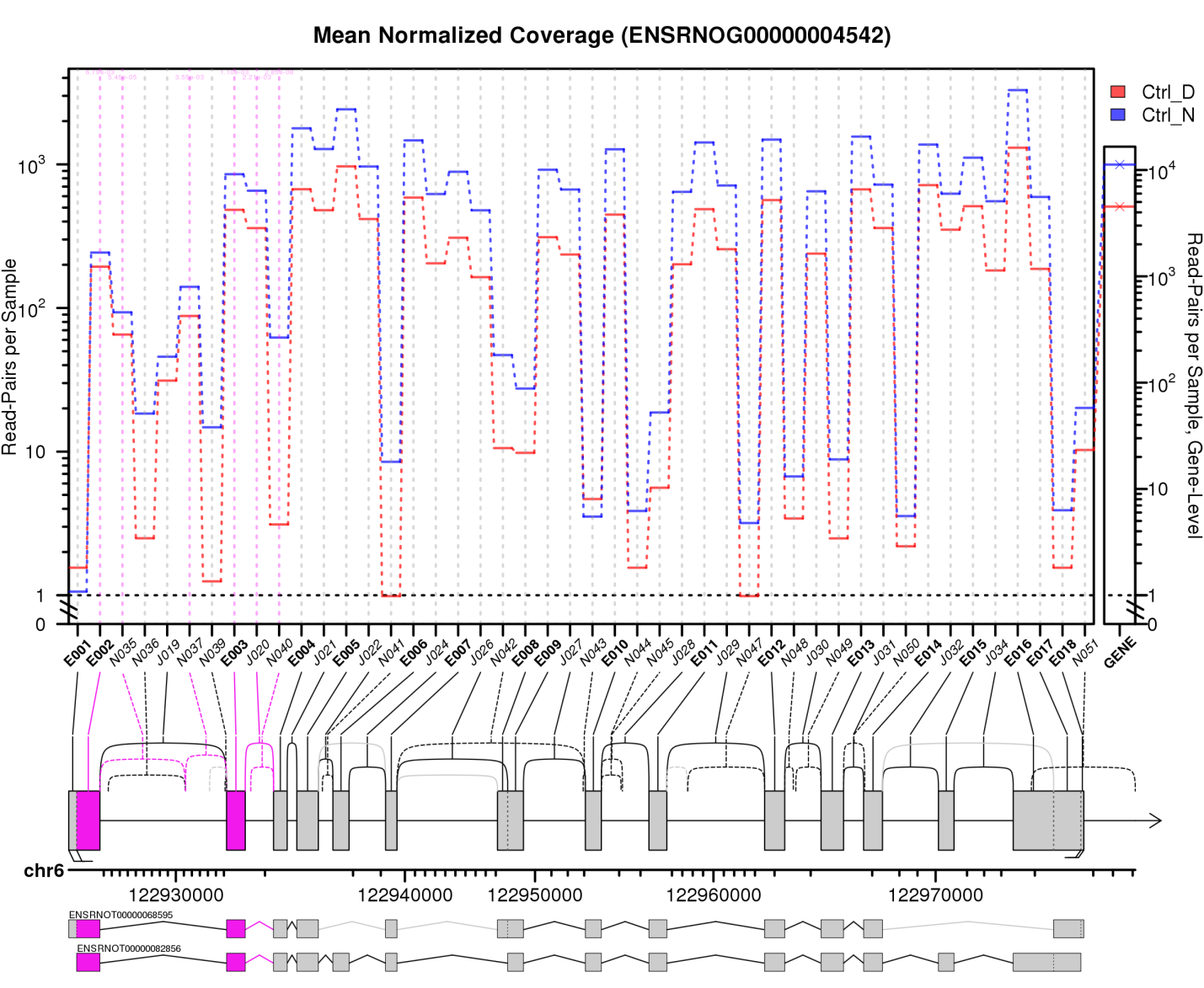
**

S2 figure: JunctionSeq results for the Ttc8 gene, control night/day experiment.

**
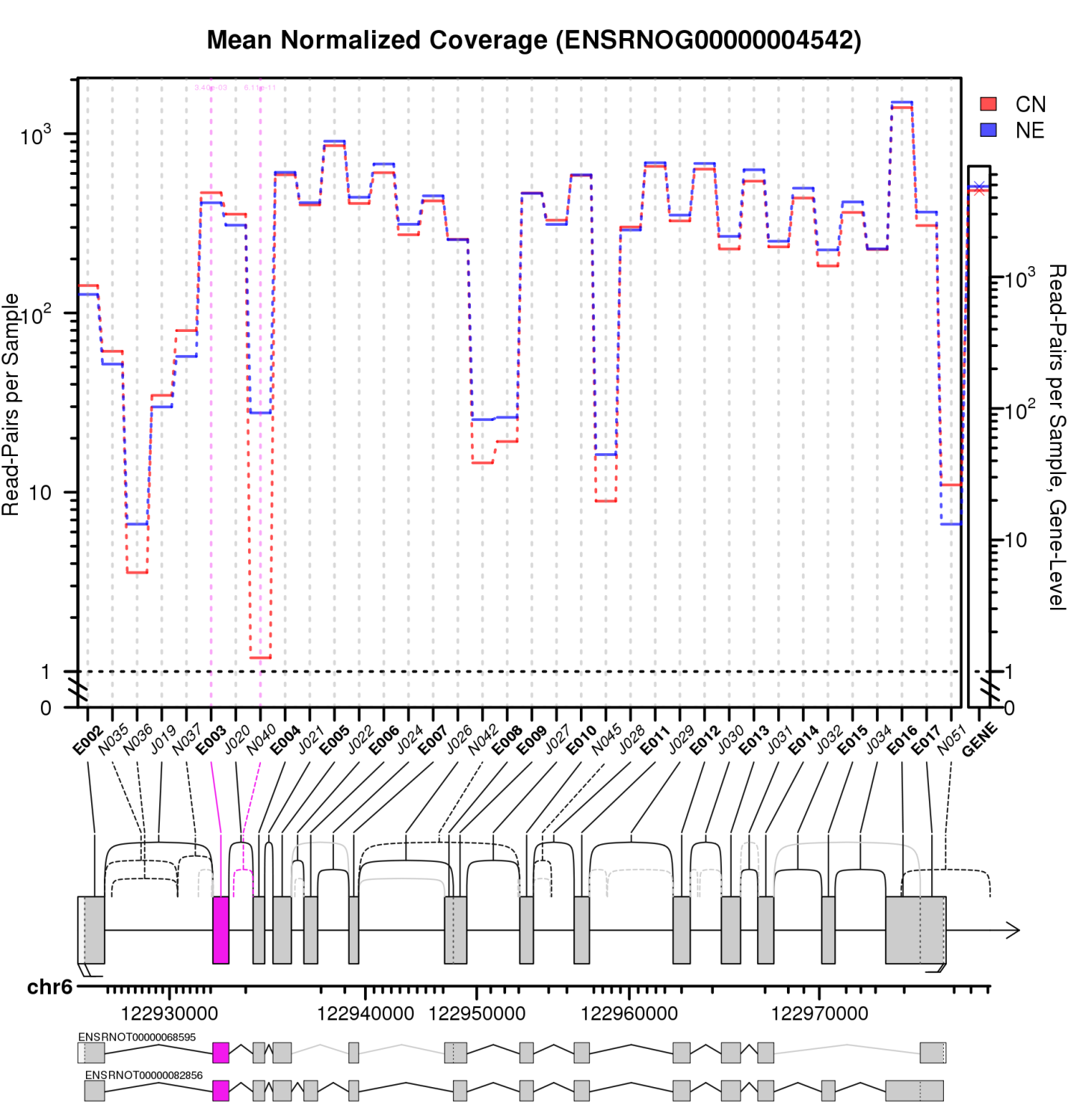
**

S3 figure: JunctionSeq results for the Ttc8 gene, Untreated vs NE experiment.

**
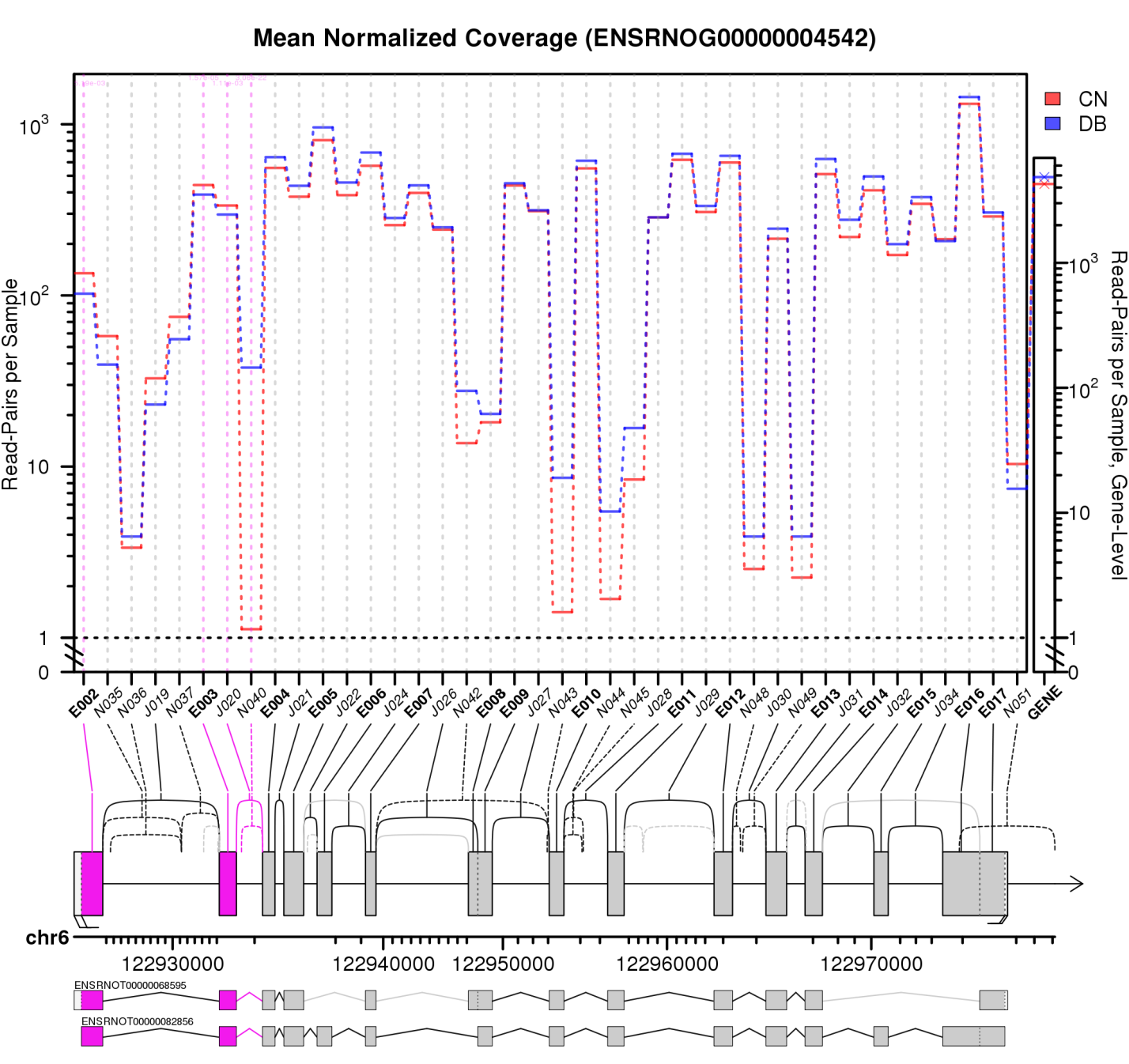
**

S4 figure: JunctionSeq results for the Ttc8 gene, Untreated vs DBcAMP experiment.

**
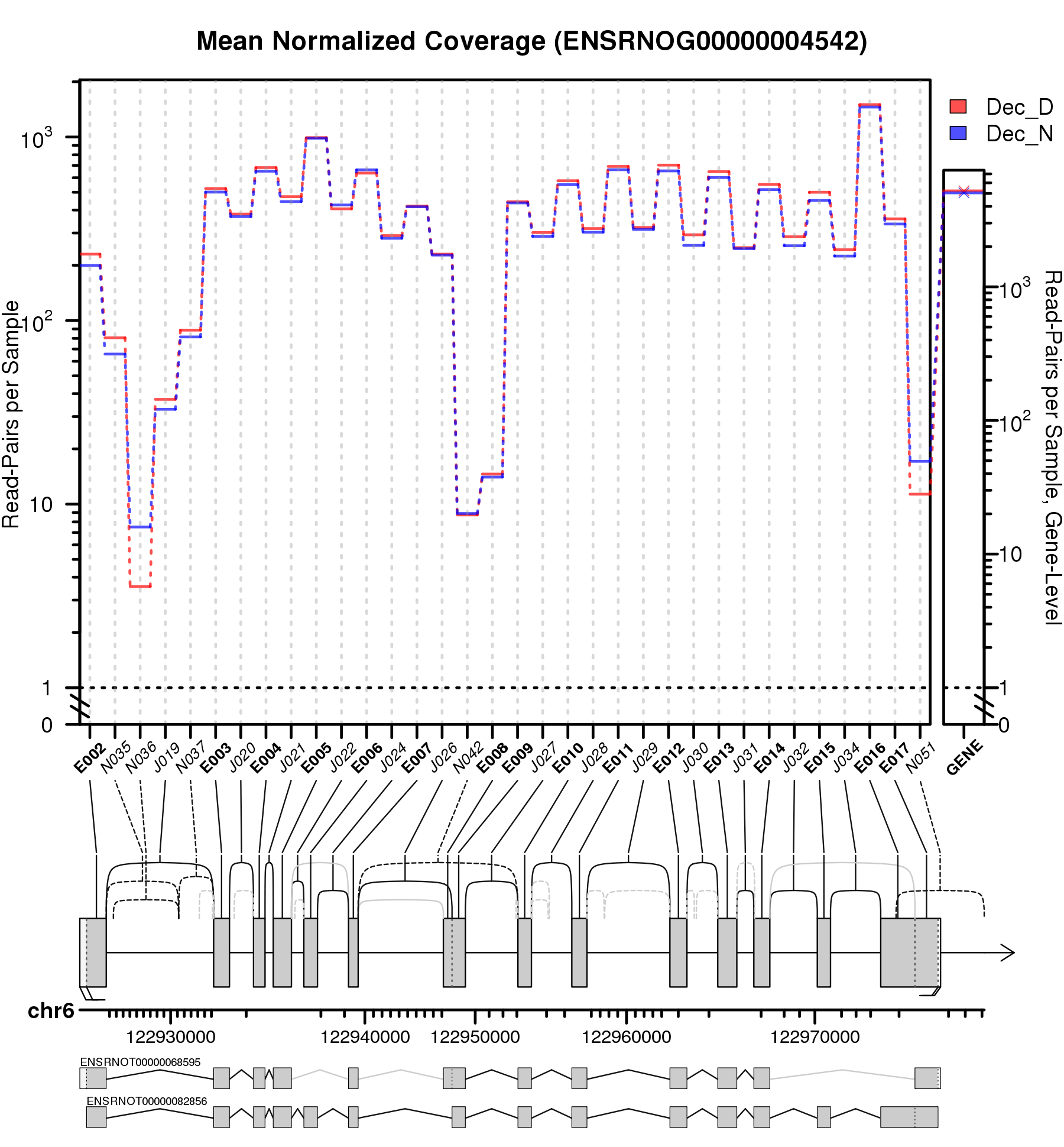
**

S5 figure: JunctionSeq results for the Ttc8 gene, DCN (Dec) night/day experiment.

**
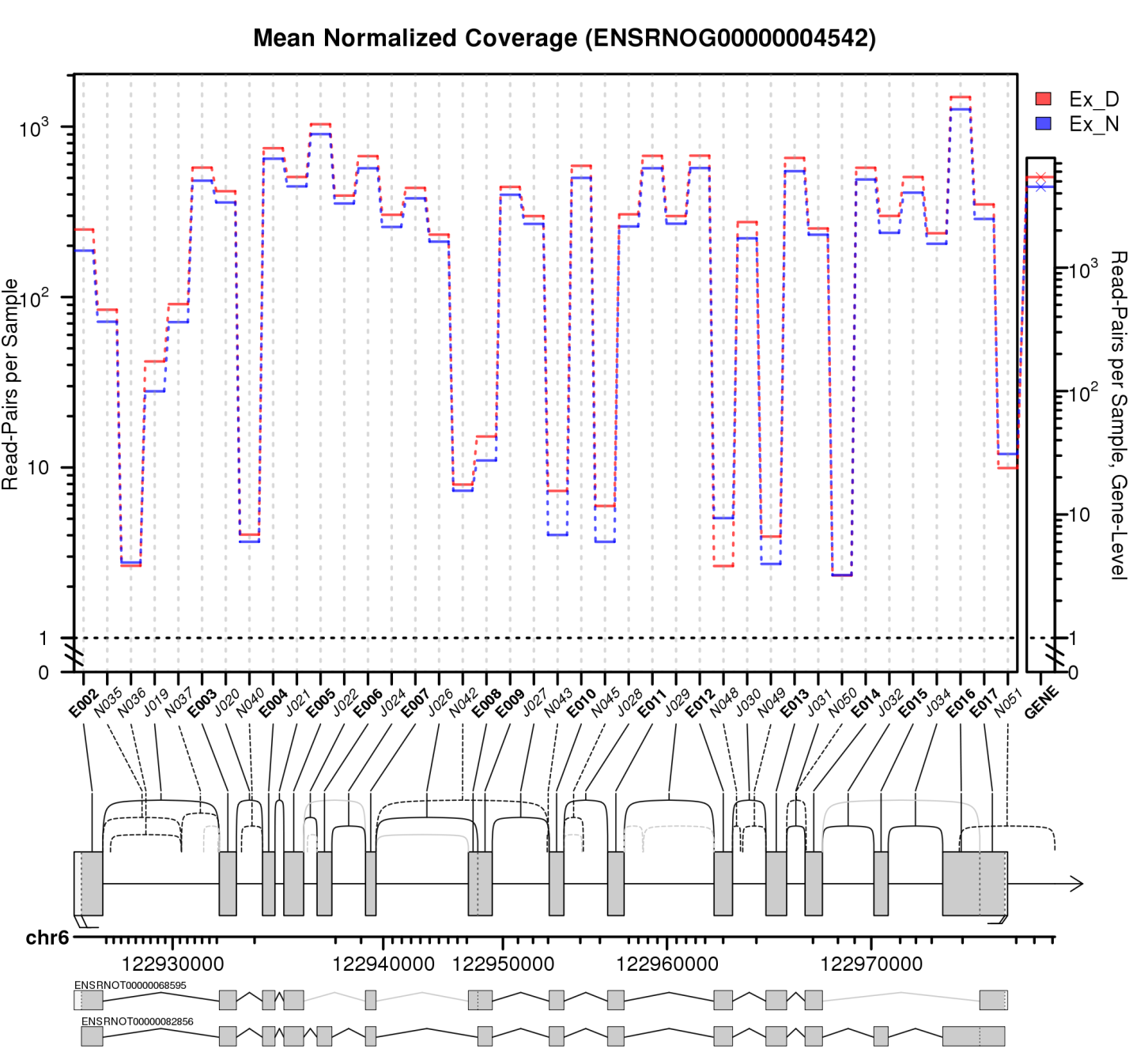
**

S6 figure: JunctionSeq results for the Ttc8 gene, SCGX (Ex) night/day experiment.


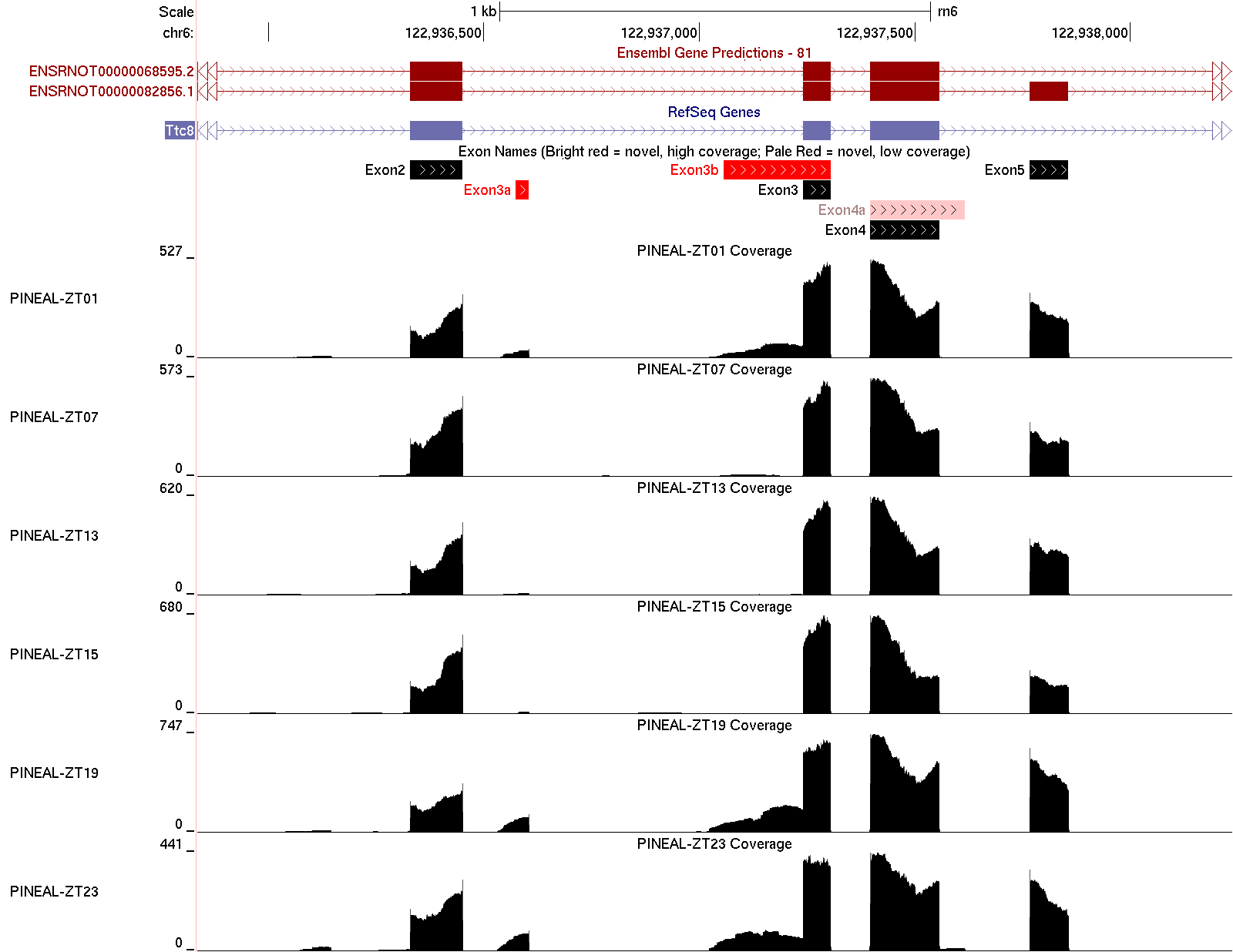


S7 figure: RNA-Seq data from the rat pineal gland sampled at various times during the day. Rats were housed in a 14:10 light:dark cycle. ZT, Zeitgeber time.


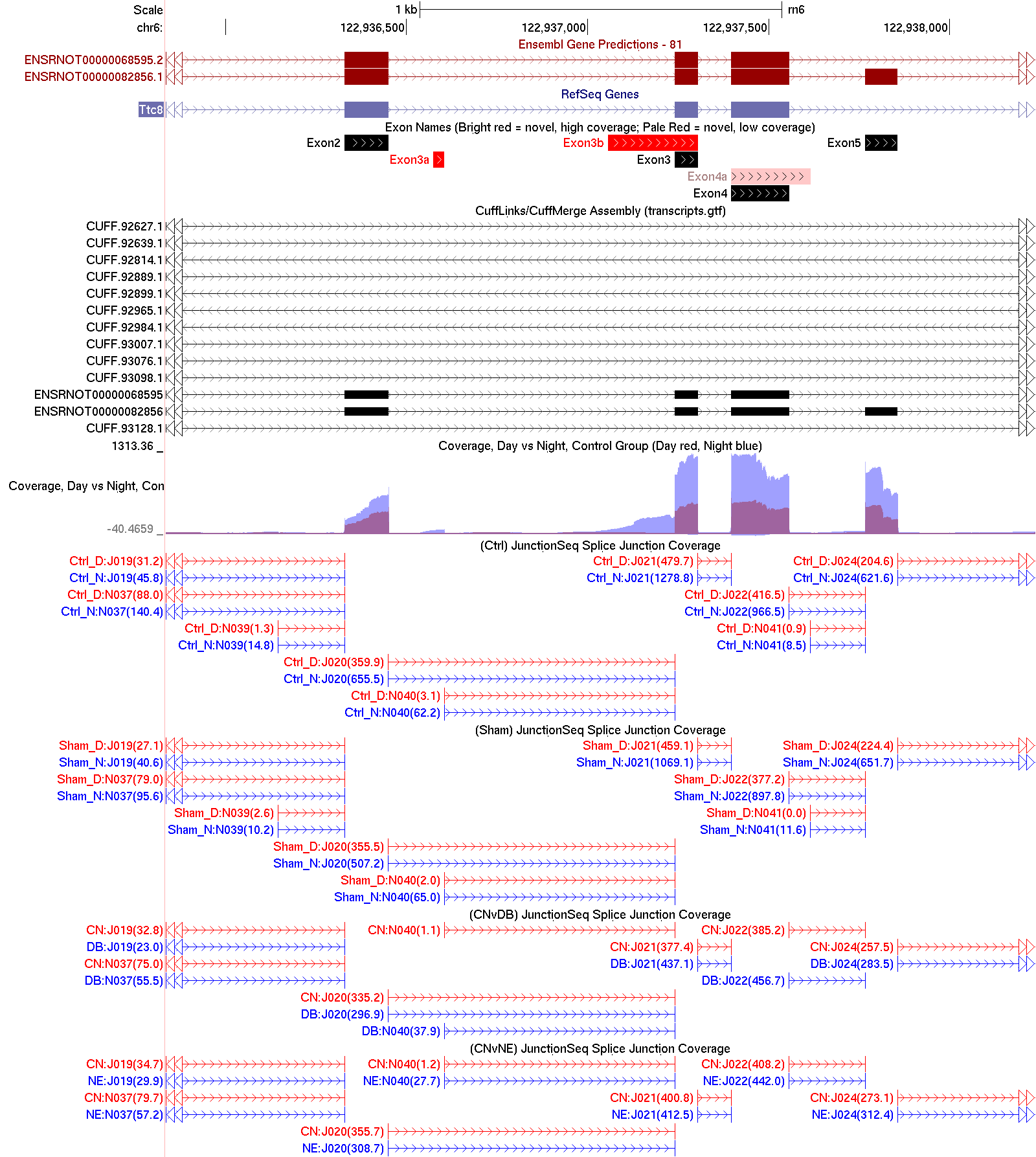


S8 figure: CuffLinks assembly for the region shown in Fig 2. As you can see, there are 30-60 read pairs per sample covering junction N040 in the innervated sample groups (Ctrl_N, Sham_N, DBcAMP and NE). However, CuffLinks does not detect any transcripts containing this junction. It also fails to detect novel start site 3b despite substantial read coverage over this region. Subsequent validation proved that these novel splice sites are real.


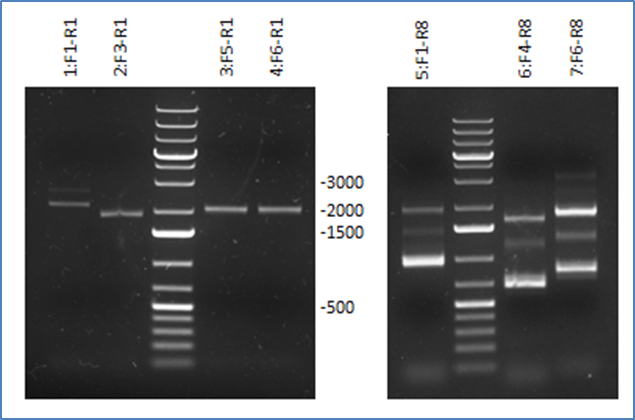


S9 figure: Images of the PCR products of reactions that were pooled for SMRT sequencing. The label above each lane indicates the reaction number and the primer pair that was used for amplification. See S2 table for primer sequences.


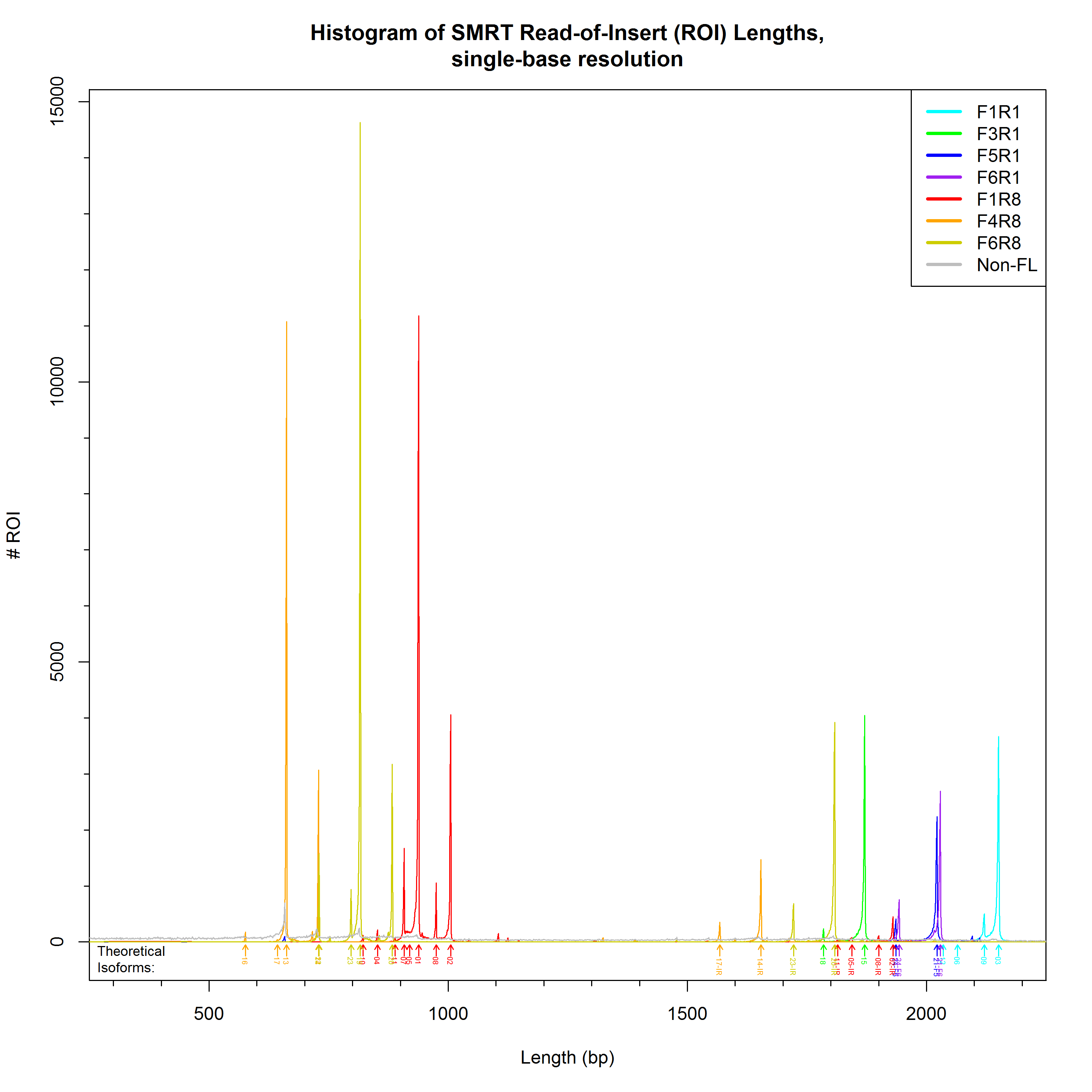


S10 figure: Read lengths from PacBio SMRT sequencing. This plot shows a base-pair-resolution histogram of the read-of-insert (ROI) length produced by the PacBio SMRT sequencing of the Ttc8 gene. The reads are separated by primer pair and drawn separately in each color. The lengths of major potential predicted isoforms are marked at the bottom, along with the potential isoform ID. Reads of insert that did not match a legal primer pair at both ends were assumed to be non-full-length (non-FL) and are plotted in gray. Note that due to the accuracy of the SMRT sequencing platform, the presence of many of the predicted isoforms can be easily recognized based only on the high number of ROI at that exact length. Also note that this effect is less pronounced for the longer isoforms, due to the fact that the ROI accuracy is inversely associated with template length.


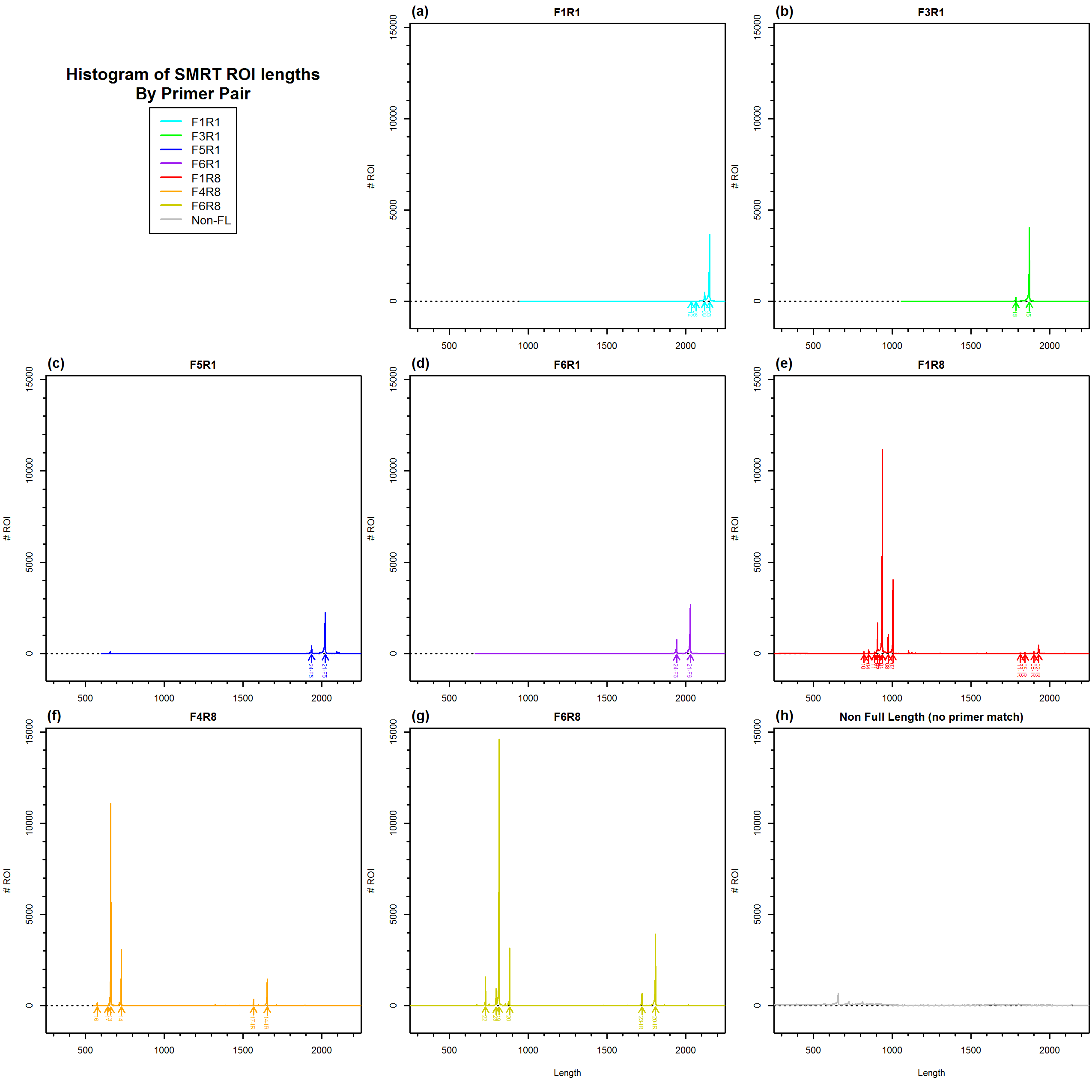


S11 figure: The same information displayed in S10 figure, separated by primer pair matching.


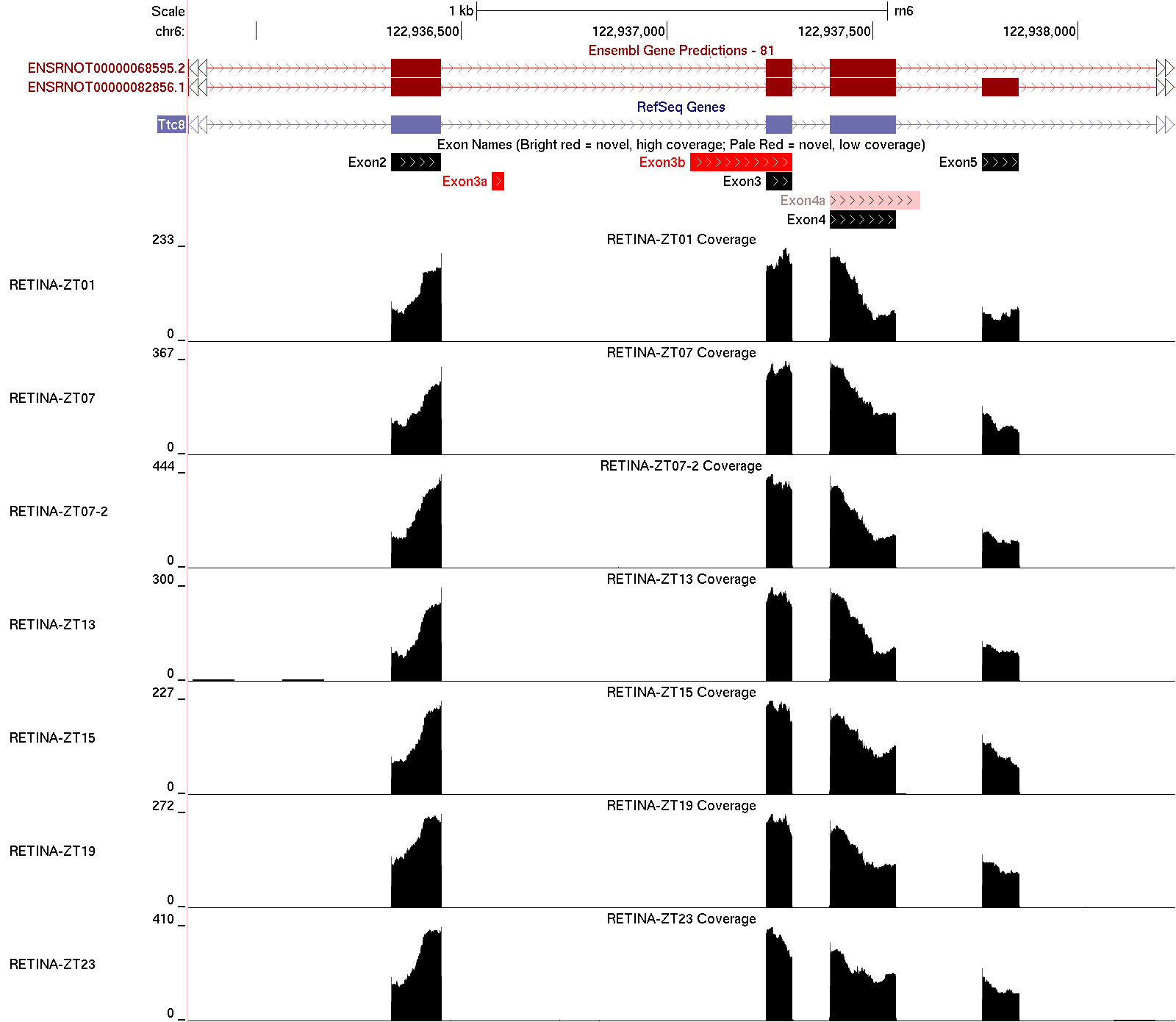


S12 figure: RNA-Seq data from the rat retina sampled at various times during the day. Rats were housed in a 14:10 light:dark cycle. Note that there are 2 samples at ZT7. Note that there are no reads covering the novel exon 3a, nor covering the region specific to novel exon 3b. ZT, Zeitgeber time.

# List of Supplemental Datasets

These supplemental files are available online.

S1 dataset: (gtf.gz) A transcript assembly GTF file generated by CuffLinks/CuffMerge (v2.0.2). These transcripts were generated via a CuffLinks run on each sample in the discovery set, followed by the use of CuffMerge to merge these individual-sample assemblies. Note that CuffLinks failed to discover any of the novel transcripts, and there are no transcripts covering any of the 17 novel splice junctions observed in the Illumina and PacBio datasets.

S2 dataset: (txt) A tab-delimited text file that describes all theoretical isoforms. It includes columns listing the ROI counts from the PacBio SMRT sequencing.

S3 dataset: (txt) A tab-delimited text file that describes each of the “major” sub-variant combinations. It includes columns listing the ROI counts from the PacBio SMRT sequencing. See also Fig 4c.

S4 dataset: (xlsx) JunctionSeq results. An excel file containing the JunctionSeq results for each analysis, for all genes that showed significant differential usage.

S5 dataset: (gff.gz) A gzip-compressed GFF file containing the JunctionSeq annotation. This includes all exonic regions and known/novel splice junctions and their unique identifiers.

S6 dataset: (xlsx) An excel spreadsheet with the 42 novel isoforms. This includes all theoretical isoforms that had one or more perfect, full-length matches in the PacBio dataset. Also included is the GenBank accession numbers for each isoform.

# References

1. Dobin A, Davis CA, Schlesinger F, Drenkow J, Zaleski C, Jha S, et al. STAR: ultrafast universal RNA-seq aligner. Bioinformatics. 2013;29(1):15-21.

2. Flicek P, Ahmed I, Amode MR, Barrell D, Beal K, Brent S, et al. Ensembl 2013. Nucleic acids research. 2013;41(Database issue):D48-55. doi: 10.1093/nar/gks1236. PubMed PMID: 23203987; PubMed Central PMCID: PMC3531136.

3. Hartley SW, Mullikin JC. QoRTs: a comprehensive toolset for quality control and data processing of RNA-Seq experiments. BMC bioinformatics. 2015;16:224. doi: 10.1186/s12859-015-0670-5. PubMed PMID: 26187896; PubMed Central PMCID: PMC4506620.

4. Care IoLARCo, Animals UoL, Resources NIoHDoR. Guide for the care and use of laboratory animals: National Academies; 1985.

5. Kilkenny C, Browne W, Cuthill IC, Emerson M, Altman DG. Animal research: reporting in vivo experiments: the ARRIVE guidelines. British journal of pharmacology. 2010;160(7):1577-9.

6. Foulkes NS, Borjigin J, Snyder SH, Sassone-Corsi P. Transcriptional control of circadian hormone synthesis via the CREM feedback loop. Proceedings of the National Academy of Sciences. 1996;93(24):14140-5.

7. Korf H-W, Schomerus C, Maronde E, Stehle J. Signal transduction molecules in the rat pineal organ: Ca 2+, pCREB, and ICER. Naturwissenschaften. 1996;83(12):535-43.

8. Schwartz WJ, Aronin N, Sassone-Corsi P. Photoinducible and rhythmic ICER-CREM immunoreactivity in the rat suprachiasmatic nucleus. Neuroscience letters. 2005;385(1):87-91. doi: 10.1016/j.neulet.2005.05.018. PubMed PMID: 15936880.

9. Kim J-S, Bailey MJ, Ho AK, Møller M, Gaildrat P, Klein DC. Daily rhythm in pineal phosphodiesterase (PDE) activity reflects adrenergic/3′, 5′-cyclic adenosine 5′-monophosphate induction of the PDE4B2 variant. Endocrinology. 2007;148(4):1475-85.

10. Borjigin J, Payne AS, Deng J, Li X, Wang MM, Ovodenko B, et al. A novel pineal night-specific ATPase encoded by the Wilson disease gene. The Journal of neuroscience. 1999;19(3):1018-26.

11. Finn RD, Clements J, Eddy SR. HMMER web server: interactive sequence similarity searching. Nucleic acids research. 2011:gkr367.

# List of Main Text Tables and Figures

Table 1: The number of genes detected in each JunctionSeq analysis at various p-value cutoffs.

Table 2: Summary information on the 18 genes detected at p-adjust < 0.0001 in all four analyses.

Table 3: Distribution of “reads of insert” (ROI) from the SMRT sequencing.

Fig 1: JunctionSeq gene profile plot for Ttc8 gene, sham night/day experiment.

Fig 2: Summary browser tracks generated by QoRTs/JunctionSeq for the region surrounding exons 2 to 5 of Ttc8.

Fig 3: All possible sub-variants detected in the Illumina RNA-Seq data.

Fig 4: Diagram of the 24 potential isoforms produced by the “major” sub-variants, with PacBio match counts.

Fig 5: Results of the start-site qPCR experiment.

Fig 6: Additional novel isoforms discovered or validated via the PacBio SMRT sequence data.
