# Supplementary figures and images for: Alternative Isoform Analysis of Ttc8 Expression in the Rat Pineal Gland Using a Multi-Platform Sequencing Approach Reveals Neural Regulation
Source: PLoS One. 2016 Sep 29;11(9):e0163590. doi: 10.1371/journal.pone.0163590 (PMC5042479; doi:10.1371/journal.pone.0163590)

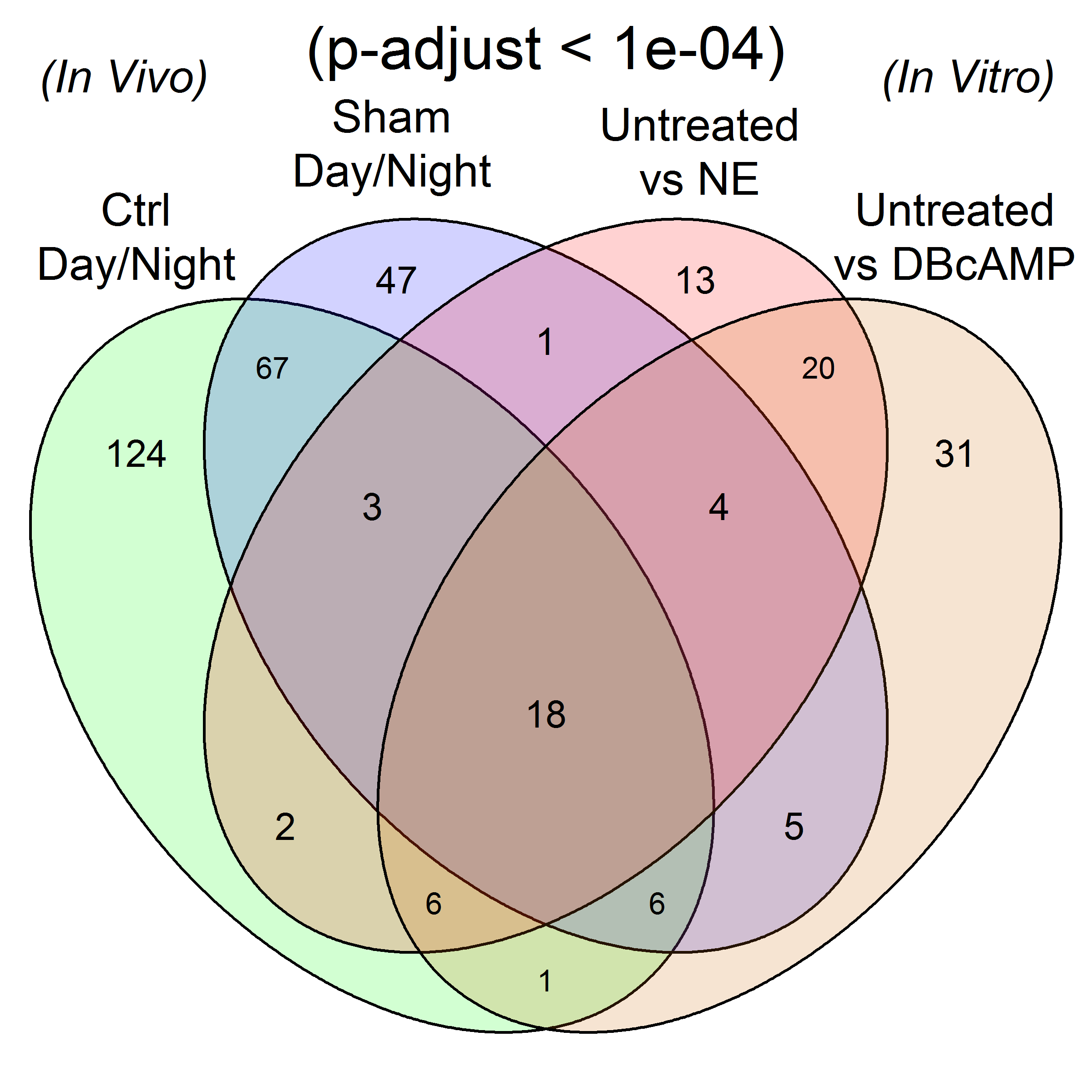

Supplement: S1 Fig — This Venn diagram displays the overlap between the genes detected as containing a differentially used feature in each of the four JunctionSeq “stimulus” analyses at the adjusted-p-value < 0.0001 level. (PNG) [file pone.0163590.s008.png]

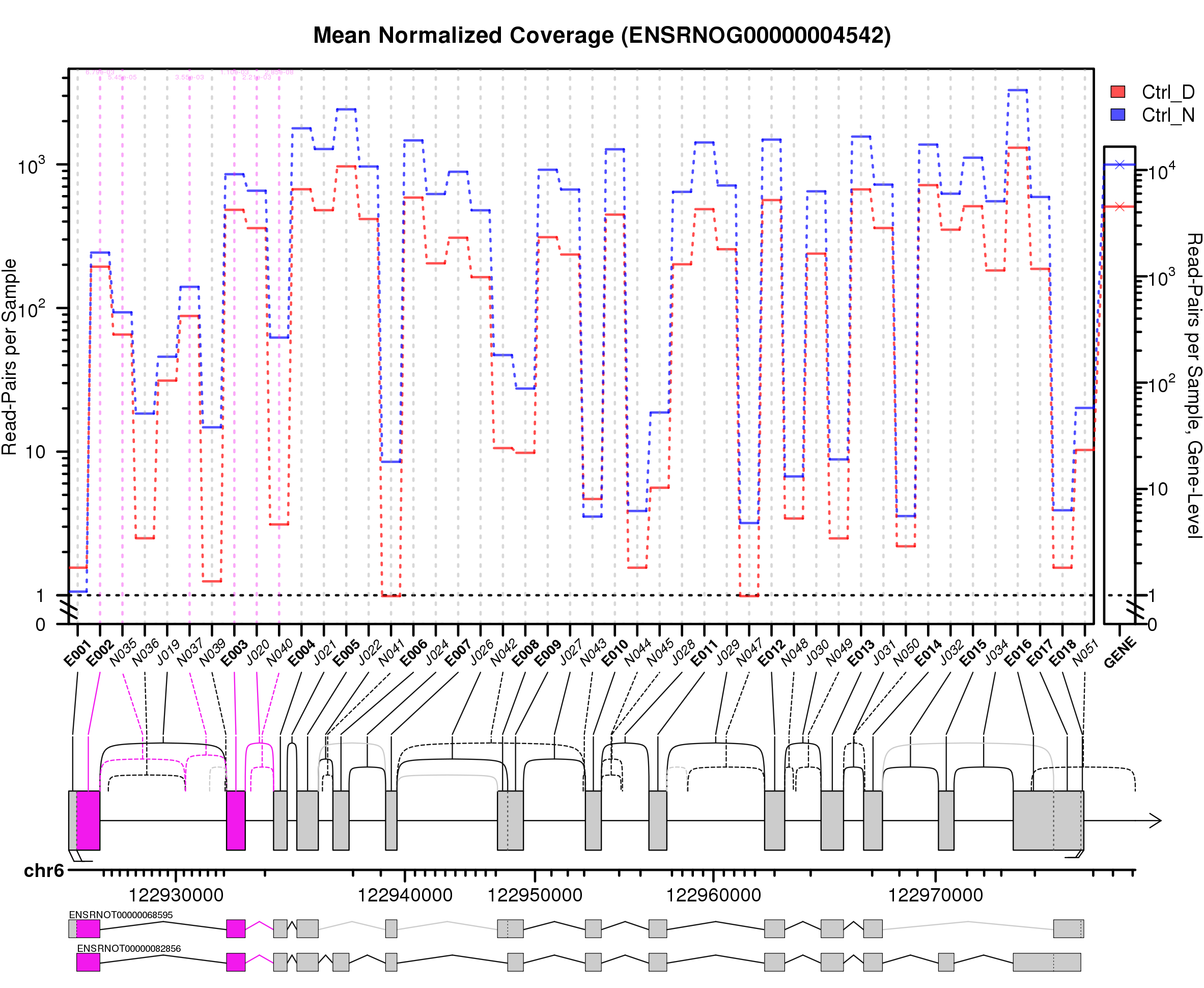

Supplement: S2 Fig — (PNG) [file pone.0163590.s009.png]

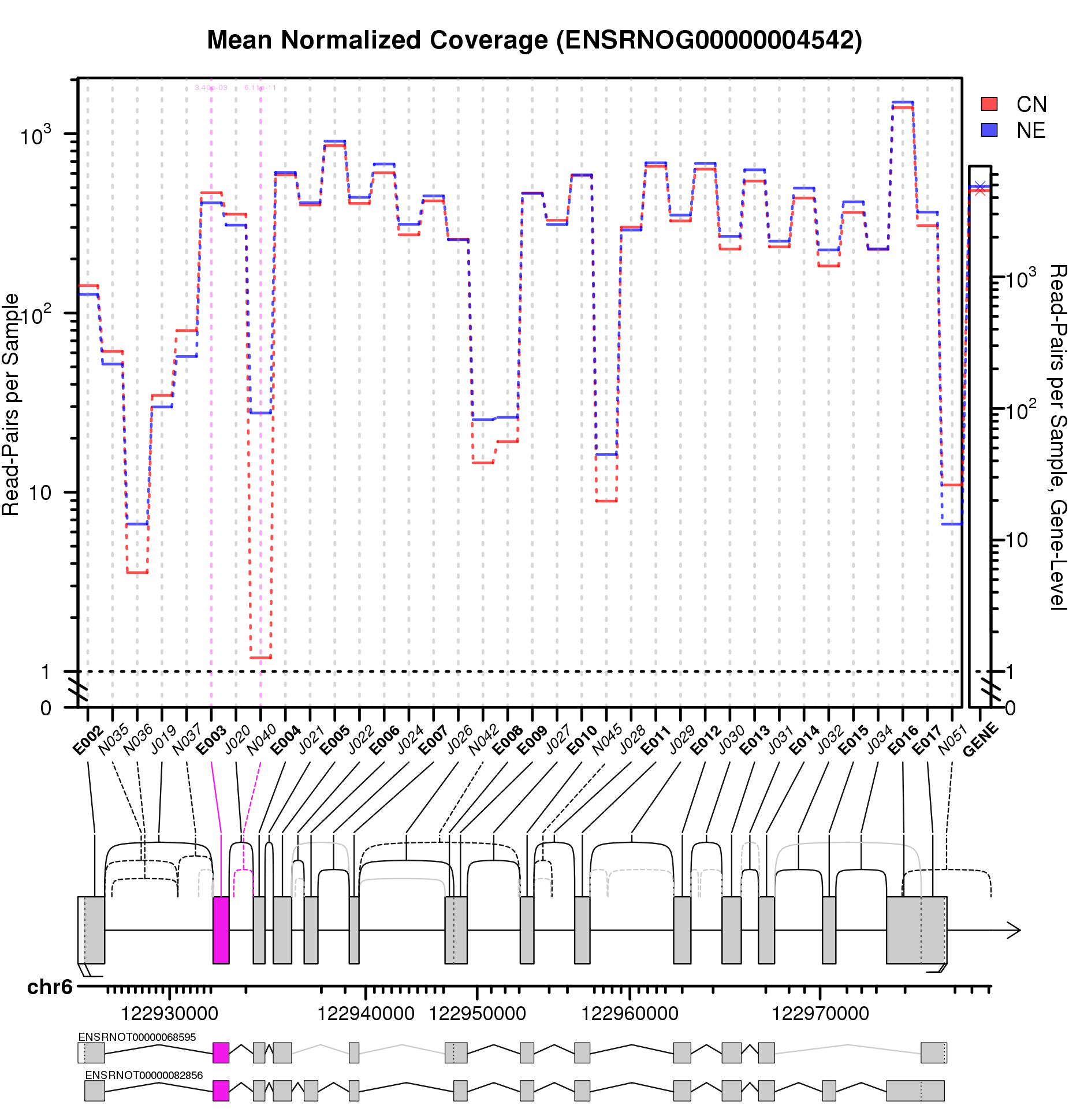

Supplement: S3 Fig — (PNG) [file pone.0163590.s010.png]

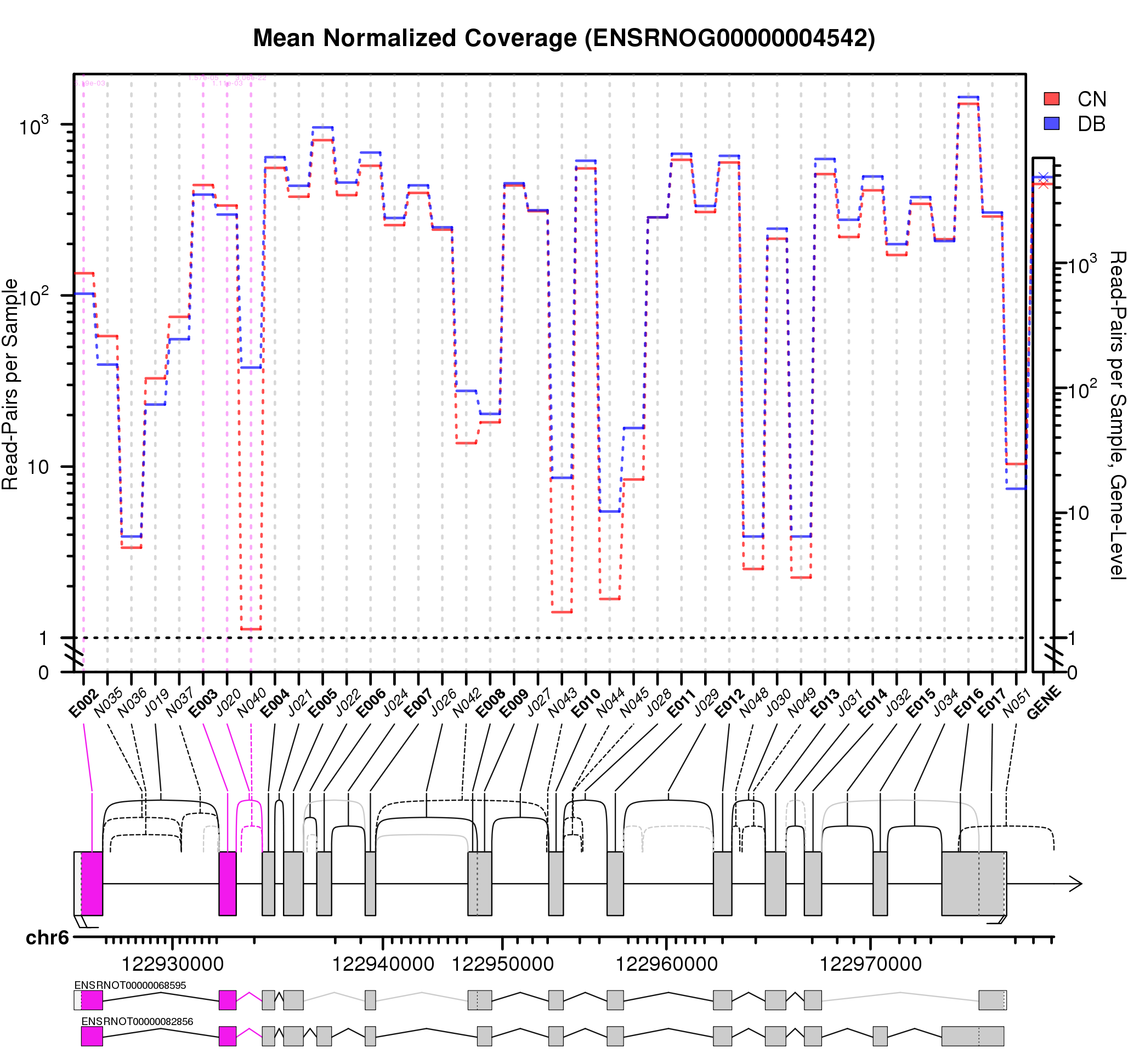

Supplement: S4 Fig — (PNG) [file pone.0163590.s011.png]

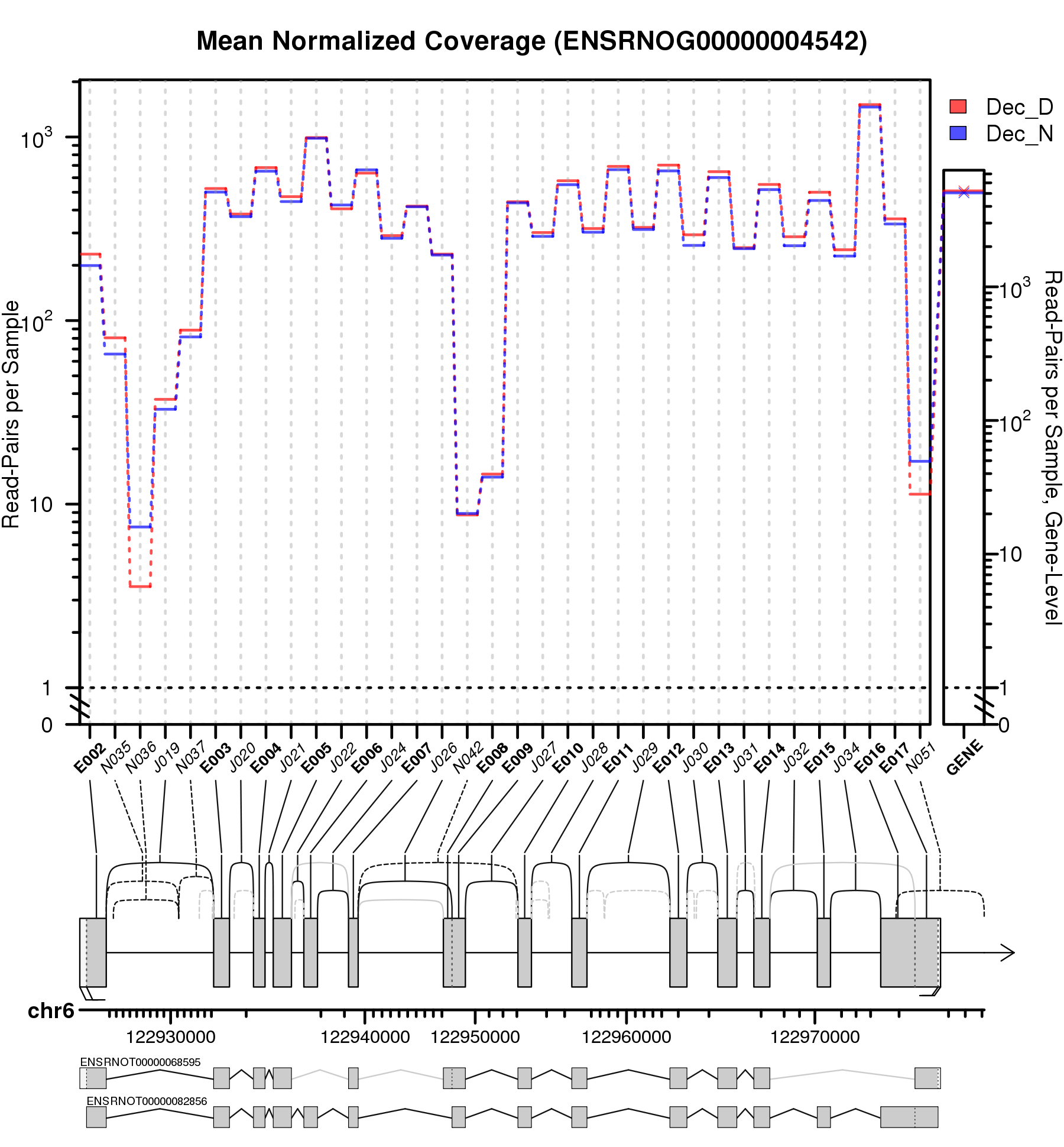

Supplement: S5 Fig — (PNG) [file pone.0163590.s012.png]

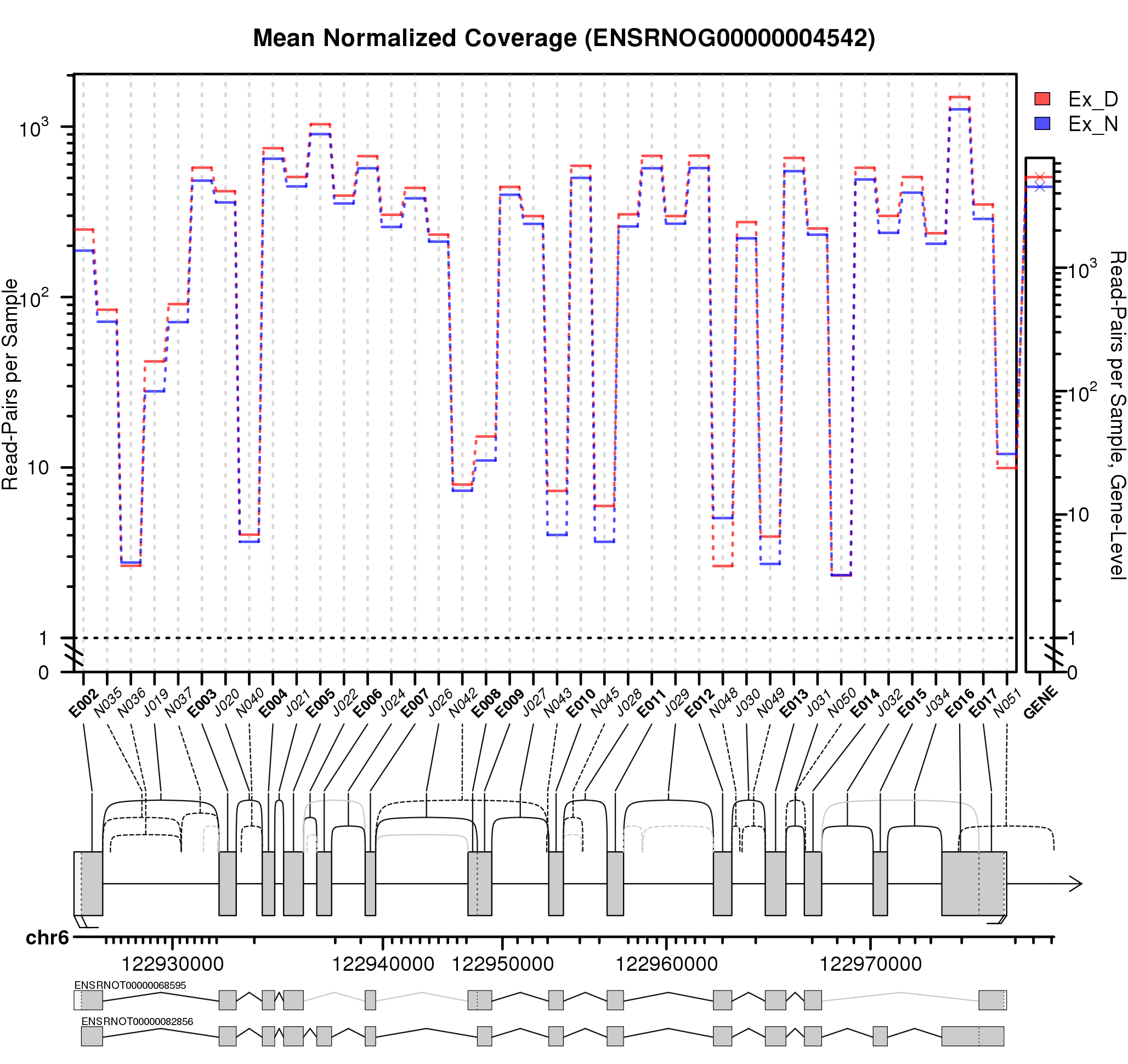

Supplement: S6 Fig — (PNG) [file pone.0163590.s013.png]

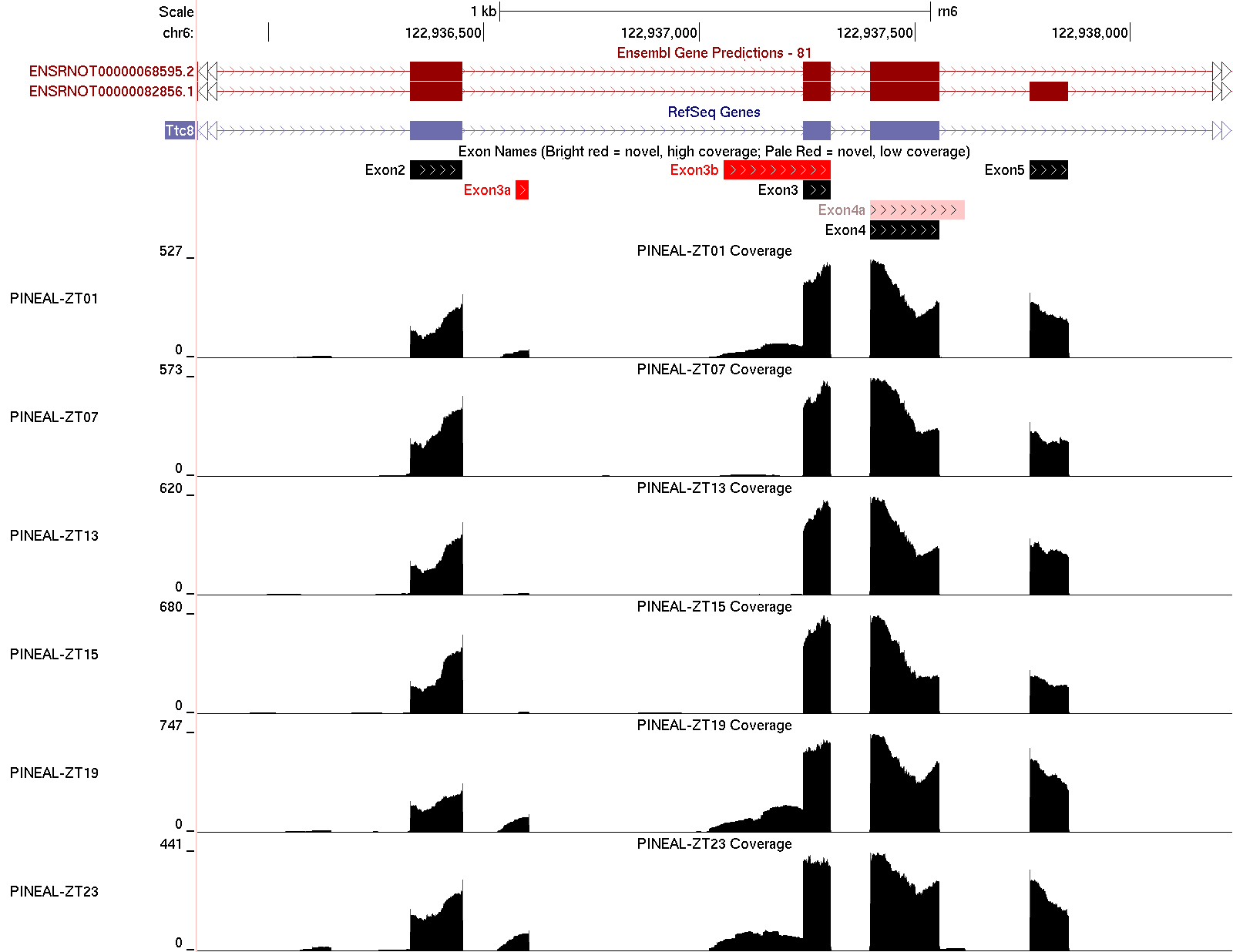

Supplement: S7 Fig — Rats were housed in a 14:10 light:dark cycle. ZT, Zeitgeber time. (PNG) [file pone.0163590.s014.png]

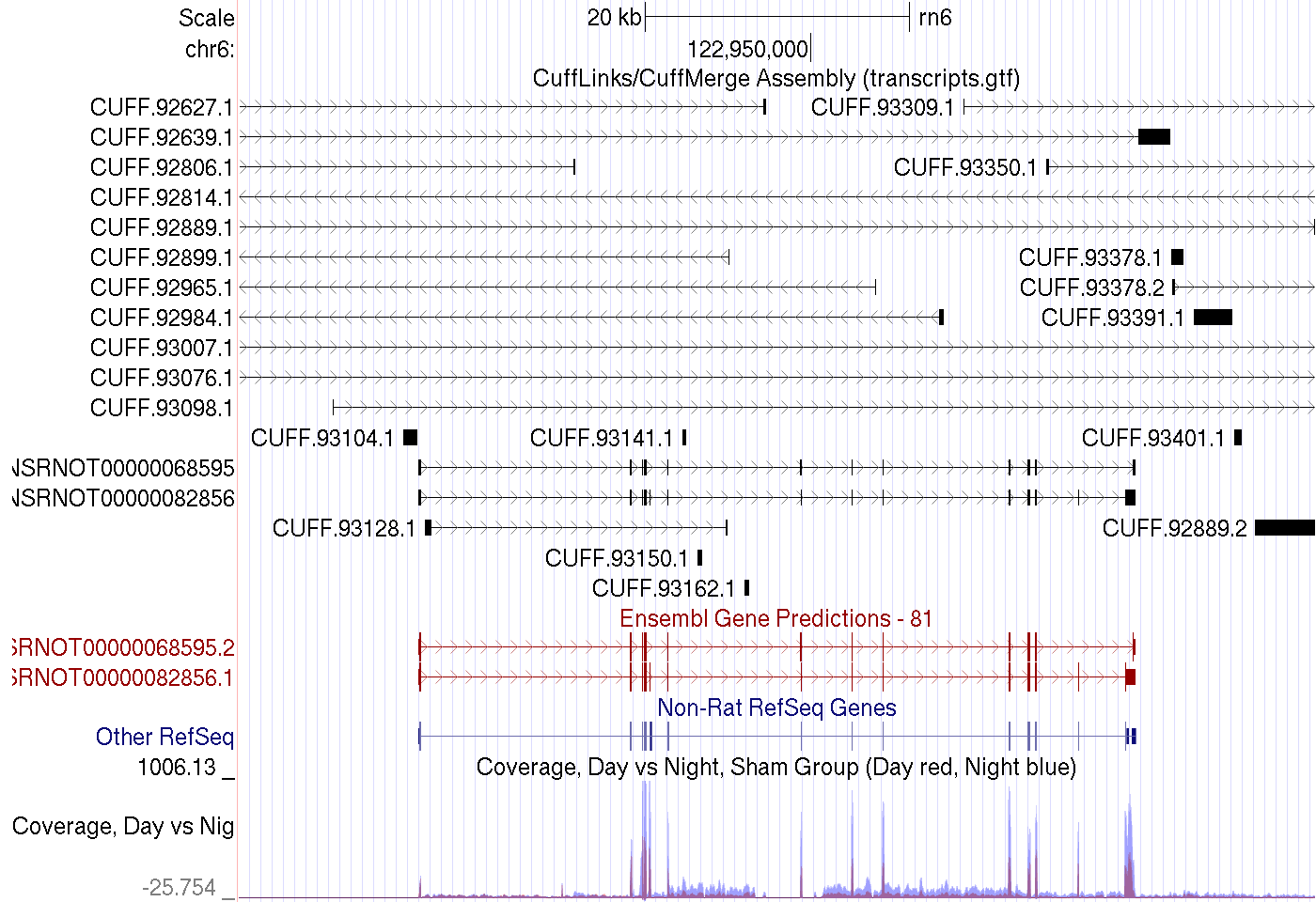

Supplement: S8 Fig — As you can see, there are 30–60 read pairs per sample covering junction N040 in the innervated sample groups (Ctrl_N, Sham_N, DBcAMP and NE). However, CuffLinks does not detect any transcripts containing this junction. It also fails to detect novel start site 3b despite substantial read coverage over this region. Subsequent validation proved that these novel splice sites are real. (PNG) [file pone.0163590.s015.png]

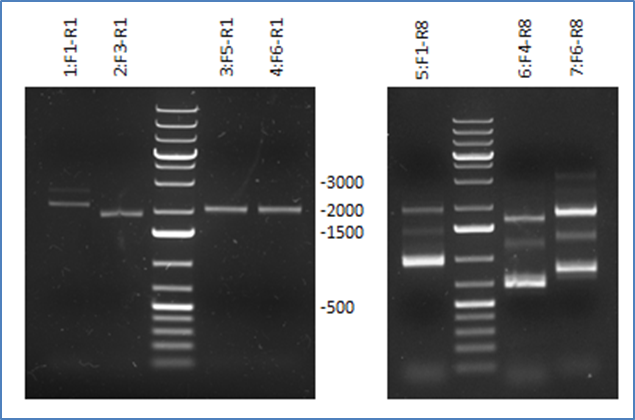

Supplement: S9 Fig — The label above each lane indicates the reaction number and the primer pair that was used for amplification. See S2 Table for primer sequences. (PNG) [file pone.0163590.s016.png]

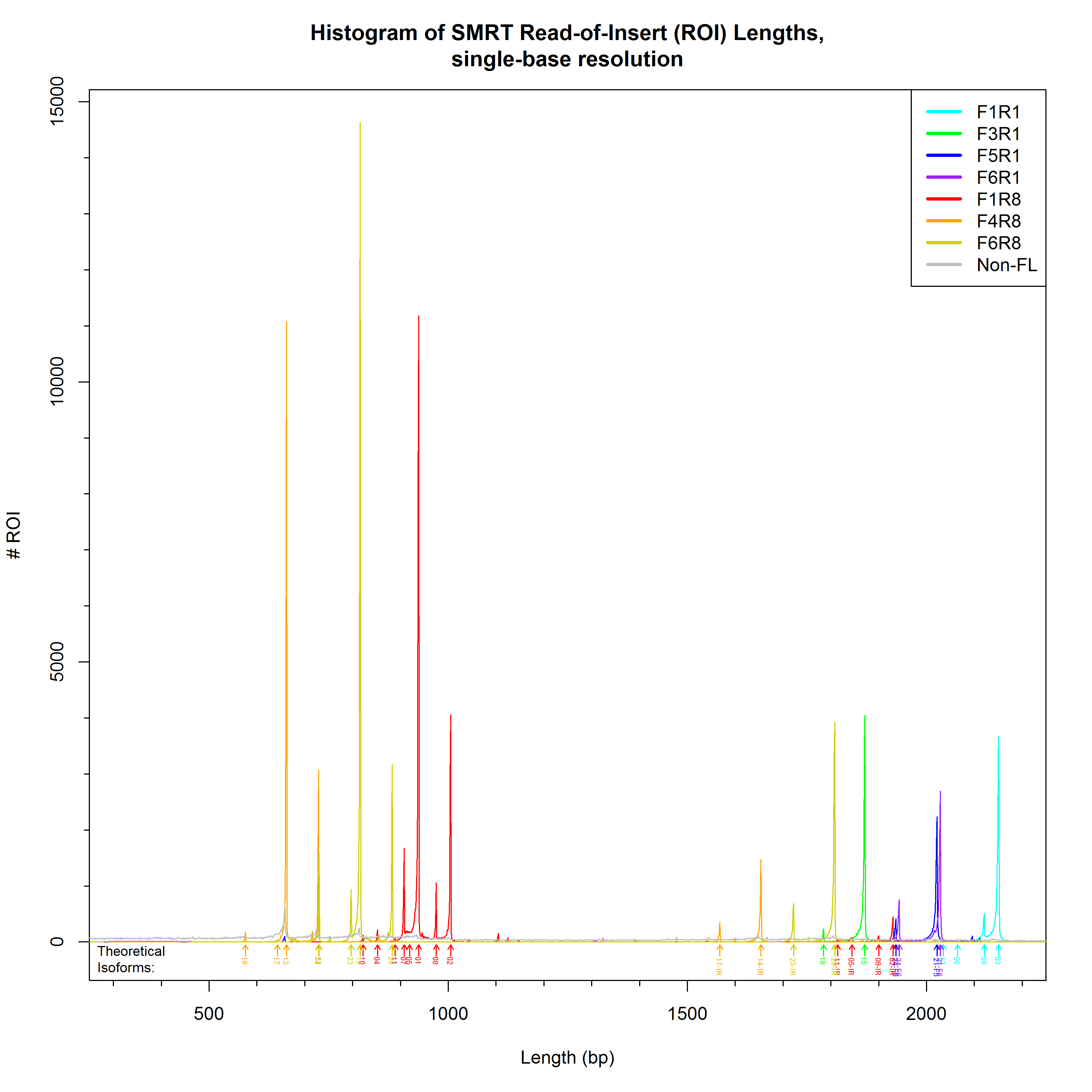

Supplement: S10 Fig — This plot shows a base-pair-resolution histogram of the read-of-insert (ROI) length produced by the PacBio SMRT sequencing of the Ttc8 gene. The reads are separated by primer pair and drawn separately in each color. The lengths of major potential predicted isoforms are marked at the bottom, along with the potential isoform ID. Reads of insert that did not match a legal primer pair at both ends were assumed to be non-full-length (non-FL) and are plotted in gray. Note that due to the accuracy of the SMRT sequencing platform, the presence of many of the predicted isoforms can be easily recognized based only on the high number of ROI at that exact length. Also note that this effect is less pronounced for the longer isoforms, due to the fact that the ROI accuracy is inversely associated with template length. (PNG) [file pone.0163590.s017.png]

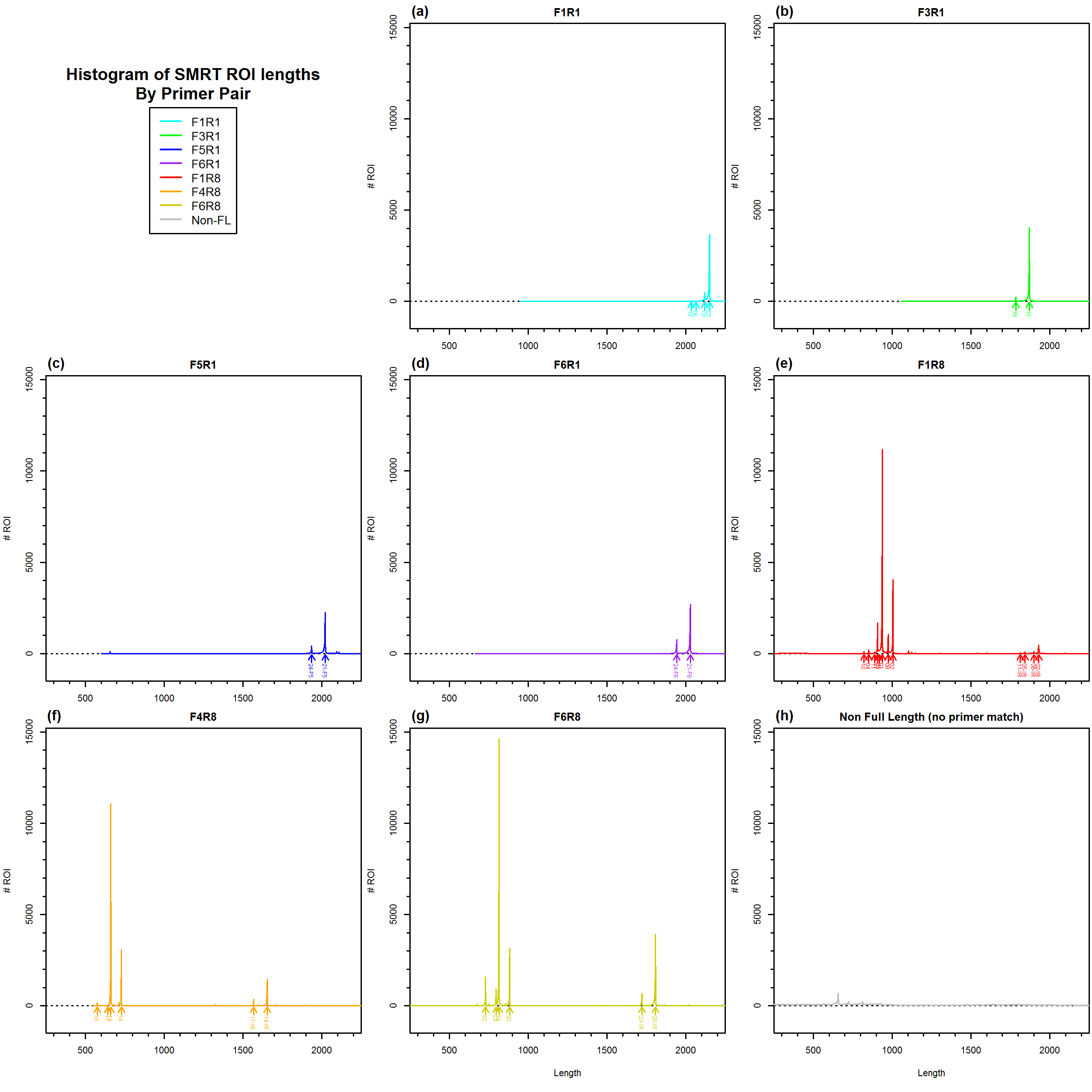

Supplement: S11 Fig — (PNG) [file pone.0163590.s018.png]

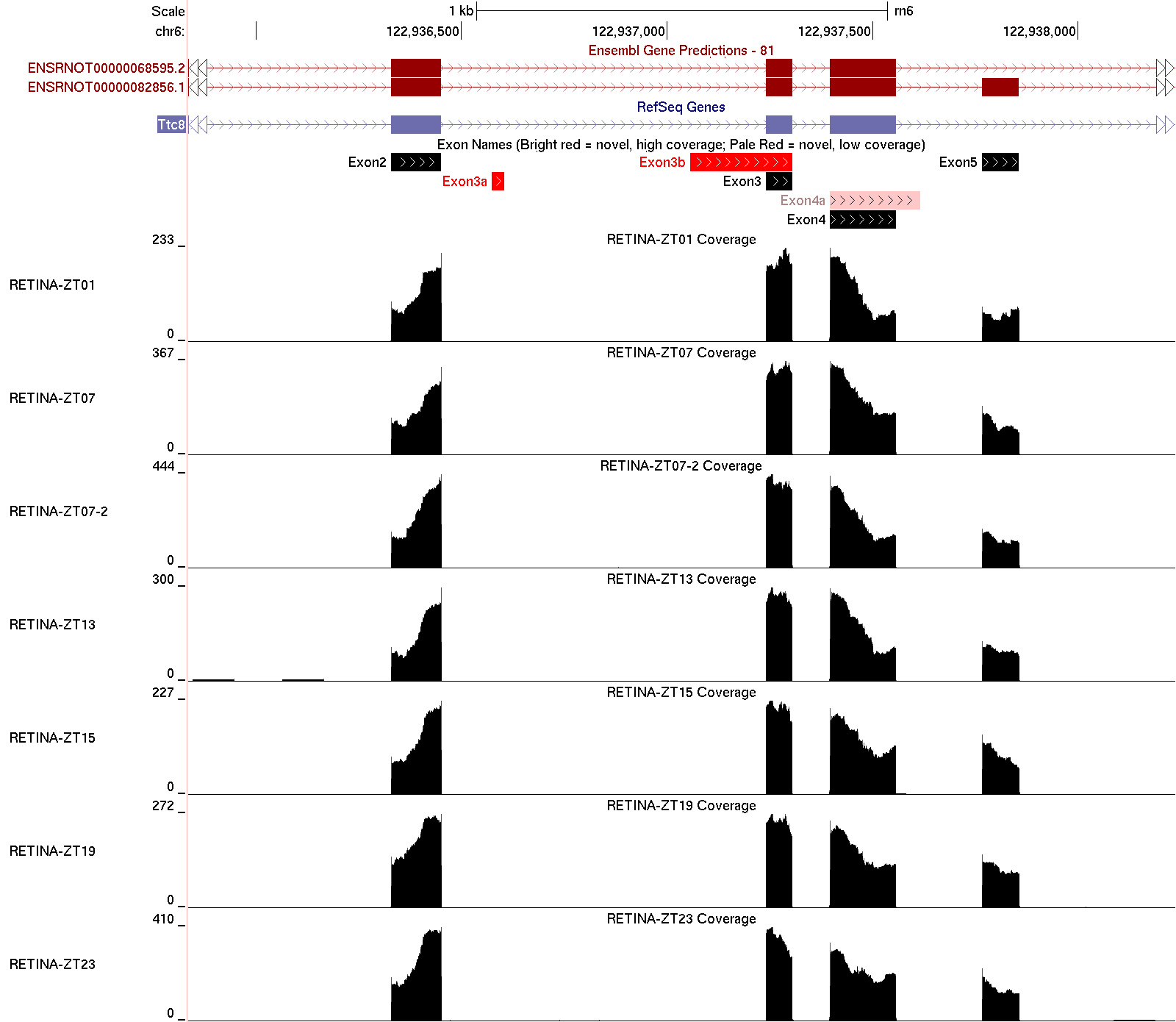

Supplement: S12 Fig — Rats were housed in a 14:10 light:dark cycle. Note that there are 2 samples at ZT7. Note that there are no reads covering the novel exon 3a, nor covering the region specific to novel exon 3b. ZT, Zeitgeber time. (PNG) [file pone.0163590.s019.png]

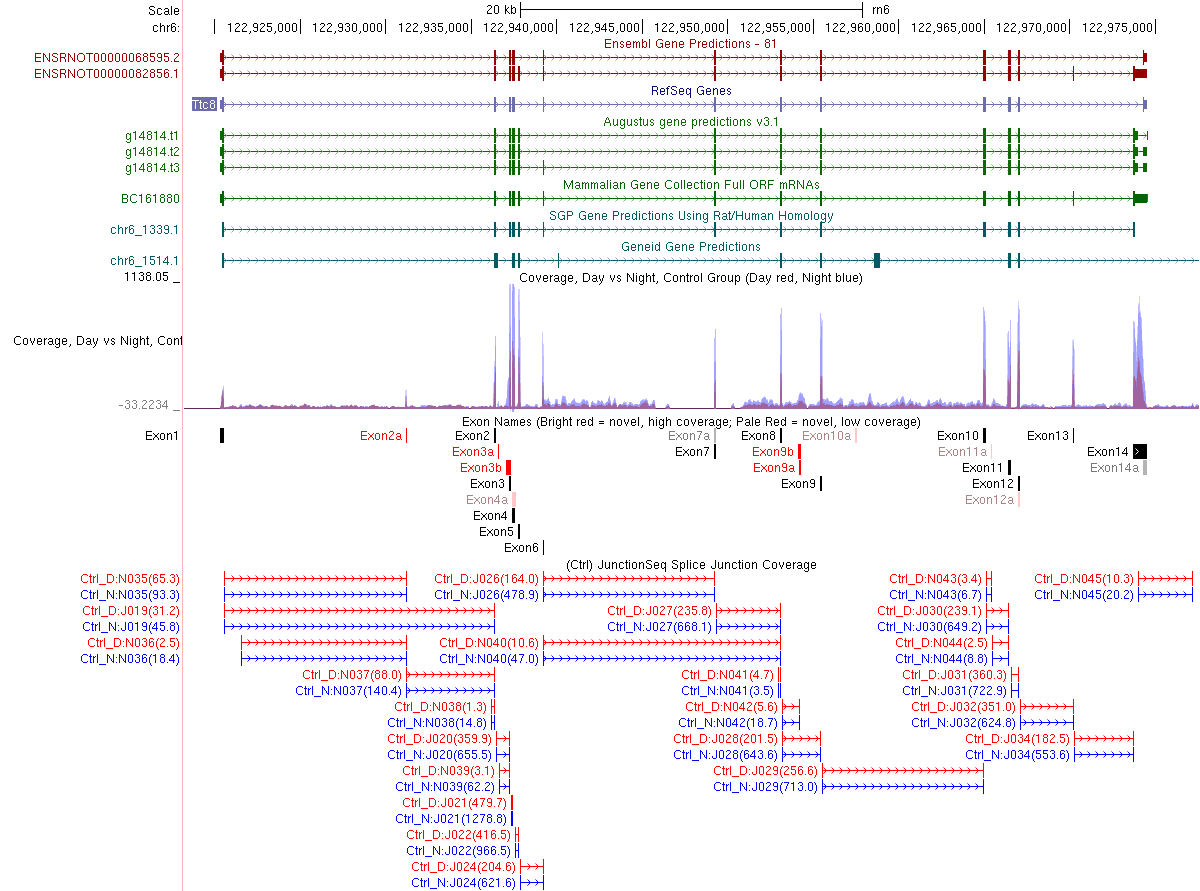

Supplement: S13 Fig — The above figure displays the gene annotations and gene predictions from Ensembl, RefSeq, Augustus, the mammalian gene collection (MGC), SGP, and geneid. Note that none of the novel exons or splice junctions appear in any of the transcript annotations. Note that the Rat Genome Database (RGD) is not included in this plot because it has not yet been lifted over to the rn6 rat genome build. See (S15 Fig) for the RGD annotation. (PNG) [file pone.0163590.s020.png]

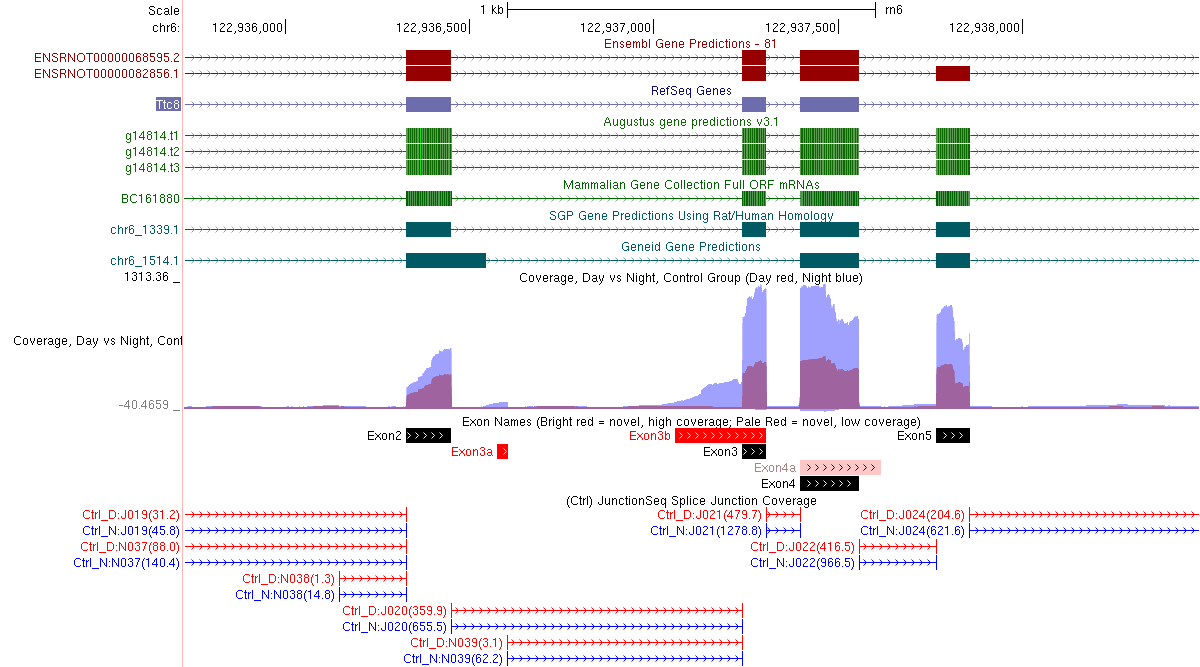

Supplement: S14 Fig — This figure is identical to the previous, except zoomed in on the region containing the major novel exons 3a and 3b. Note that none of the novel exons or splice junctions appear in any of the transcript annotations. (PNG) [file pone.0163590.s021.png]

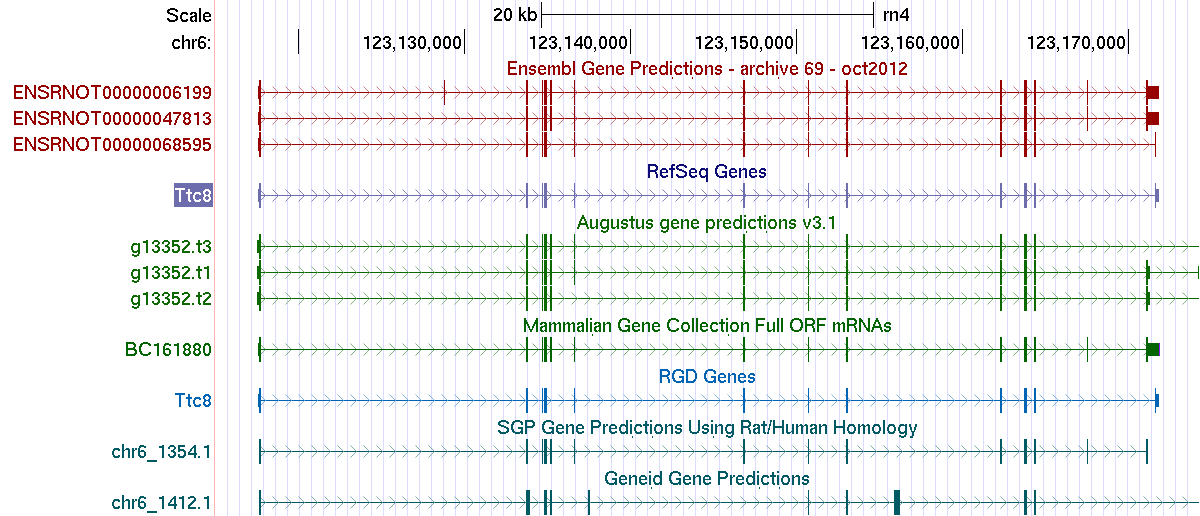

Supplement: S15 Fig — Note that this assembly includes the RGD curated gene database. Additionally, note the presence of transcript ENSRNOT00000006199 in the ensembl annotation (release 69), which was absent in subsequent ensembl releases. This transcript includes one of the “novel” exons detected in our analyses, exon 2a. The transcript appears to have been lost in the liftover to rn5 (possibly due to a nearby gap in the rn5 genome build), and was never re-added in subsequent releases. Note that none of the other novel exons or splice junctions appear in any of the transcript annotations. (PNG) [file pone.0163590.s022.png]
