# Supplementary material for: Alternative Isoform Analysis of Ttc8 Expression in the Rat Pineal Gland Using a Multi-Platform Sequencing Approach Reveals Neural Regulation
Source: PLoS One. 2016 Sep 29;11(9):e0163590. doi: 10.1371/journal.pone.0163590 (PMC5042479; doi:10.1371/journal.pone.0163590)
Supplement: S1 Table — These primers were used to confirm and quantitate the differential expression of the three start sites of the Ttc8 gene. The first primer pair addresses the annotated Ttc8 start site, and primer pairs 2 and 3 address the two major novel start sites discovered in the Illumina RNA-Seq data. (DOCX) [file pone.0163590.s023.docx]

S1 table: qPCR primer pairs used in the quantitation of start site usage. These primers were used to confirm and quantitate the differential expression of the three start sites of the Ttc8 gene. The first primer pair addresses the annotated Ttc8 start site, and primer pairs 2 and 3 address the two major novel start sites discovered in the Illumina RNA-Seq data.

| **Reaction** | **Primer Pair** | | **Primer Sequence** | |
| --- | --- | --- | --- | --- |
|  | **Forward** | **Reverse** | **Forward** | **Reverse** |
| 1 | F14 (exon 2) | R7 (exon 3) | CGTGGACCAGGAAGGGATTG | GACAGCTTGAGTCGGTCCTC |
| 2 | F4 (exon 3a) | R7 (exon 3) | GATACGGCTTTGCTGGCGAT | GACAGCTTGAGTCGGTCCTC |
| 3 | F16 (exon 3b) | R7 (exon 3) | TCTTTCCCAGCCCAGTACAAG | GACAGCTTGAGTCGGTCCTC |
| 4 | Gapdh-F1 | Gapdh-R1 | TGGTGAAGGTCGGTGTGAACGGAT | TCCATGGTGGTGAAGACGCCAGTA |
