# Supplementary material for: Alternative Isoform Analysis of Ttc8 Expression in the Rat Pineal Gland Using a Multi-Platform Sequencing Approach Reveals Neural Regulation
Source: PLoS One. 2016 Sep 29;11(9):e0163590. doi: 10.1371/journal.pone.0163590 (PMC5042479; doi:10.1371/journal.pone.0163590)
Supplement: S2 Table — These primers were used to amplify the transcripts of the Ttc8 gene prior to sequencing with the PacBio SMRT platform. Note that primers F3 and F4 differ by only 4 bases, and primers F5 and F6 differ by only 6. (DOCX) [file pone.0163590.s024.docx]

S2 table: PCR primer pairs used for PacBio SMRT sequencing. These primers were used to amplify the transcripts of the Ttc8 gene prior to sequencing with the PacBio SMRT platform. Note that primers F3 and F4 differ by only 4 bases, and primers F5 and F6 differ by only 6.

| # | **Primer Pair** | | **Primer Sequence** | | **Genomic Position** | |
| --- | --- | --- | --- | --- | --- | --- |
|  | **Forward** | **Reverse** | **Forward** | **Reverse** | **Start** | **End** |
| 1 | F1 (exon1) | R1 (exon14 ) | CACAGGACCTTTGAGCTCGT | TCTGACCTAAGTTTTCAACGATAGC | 122920371 | 122974377 |
| 2 | F3 (exon3a) | R1 (exon14) | CGGCTTTGCTGGCGATTTAT | TCTGACCTAAGTTTTCAACGATAGC | 122936579 | 122974377 |
| 3 | F5 (exon3b) | R1 (exon14) | ATGCCACCAAACAGAGCAGT | TCTGACCTAAGTTTTCAACGATAGC | 122937064 | 122974377 |
| 4 | F6 (exon3b) | R1 (exon14) | CAGGTTCATGCCACCAAACAG | TCTGACCTAAGTTTTCAACGATAGC | 122937057 | 122974377 |
| 5 | F1 (exon1) | R8 (exon9a) | CACAGGACCTTTGAGCTCGT | TGTGCCTGGTAACCTTGGAG | 122920371 | 122954254 |
| 6 | F4 (exon3a) | R8 (exon9a) | GATACGGCTTTGCTGGCGAT | TGTGCCTGGTAACCTTGGAG | 122936575 | 122954254 |
| 7 | F6 (exon3b) | R8 (exon9a) | CAGGTTCATGCCACCAAACAG | TGTGCCTGGTAACCTTGGAG | 122937057 | 122954254 |
