# Supplementary material for: Alternative Isoform Analysis of Ttc8 Expression in the Rat Pineal Gland Using a Multi-Platform Sequencing Approach Reveals Neural Regulation
Source: PLoS One. 2016 Sep 29;11(9):e0163590. doi: 10.1371/journal.pone.0163590 (PMC5042479; doi:10.1371/journal.pone.0163590)
Supplement: S3 Table — In some cases additional size selection and amplification was necessary, as per the PacBio SMRT protocol. This table lists the size windows, primers, cycle counts, and total yield. (DOCX) [file pone.0163590.s025.docx]

S3 table: Additional size-selected amplification for PacBio sequencing. In some cases additional size selection and amplification was necessary, as per the PacBio SMRT protocol. This table lists the size windows, primers, cycle counts, and total yield.

| **Fraction Number** | **Amplicon Size Range** | **PCR Primers** | **Number of Cycles** | **Total Yield** |
| --- | --- | --- | --- | --- |
| 1 | 600-1200 bp | None | 0 | 169 ng |
| 2 | 1200-2200 bp | F1/R1  F3/R1  F5/R1  F6/R1  F1/R8  F4/R8  F6/R8 | 6  6  6  6  6  6  6 | 600 ng |
| 3 | ~3000 bp | F1/R1  F6/R8 | 12  12 | 1584 ng |
