# Supplementary material for: Alternative Isoform Analysis of Ttc8 Expression in the Rat Pineal Gland Using a Multi-Platform Sequencing Approach Reveals Neural Regulation
Source: PLoS One. 2016 Sep 29;11(9):e0163590. doi: 10.1371/journal.pone.0163590 (PMC5042479; doi:10.1371/journal.pone.0163590)
Supplement: S4 Table — These are the counts for ROI that match one of the 24 potential major isoforms (see Fig 3 and Fig 4). (DOCX) [file pone.0163590.s026.docx]

S4 table: Read-of-Insert counts for the 24 “major sub-variant” potential isoforms. These are the counts for ROI that match one of the 24 potential major isoforms (see Fig 3 and Fig 4).

| **Isoform**  **ID** | **Primer**  **Pair** | **Length** | **# Perfect Match** | **# Align**  **Match** | **Total**  **Match** |
| --- | --- | --- | --- | --- | --- |
| 01 | F1-R8 | 938 | 10390 | 10328 | 20718 |
| 02 | F1-R8 | 1005 | 3695 | 3753 | 7448 |
| 03 | F1-R1 | 2151 | 1630 | 9785 | 11415 |
| 04 | F1-R8 | 852 | 202 | 184 | 386 |
| 05 | F1-R8 | 919 | 3 | 1 | 4 |
| 06 | F1-R1 | 2065 | 0 | 1 | 1 |
| 07 | F1-R8 | 908 | 1422 | 2100 | 3522 |
| 08 | F1-R8 | 975 | 917 | 1206 | 2123 |
| 09 | F1-R1 | 2121 | 153 | 1490 | 1643 |
| 10 | F1-R8 | 822 | 111 | 128 | 239 |
| 11 | F1-R8 | 889 | 70 | 57 | 127 |
| 12 | F1-R1 | 2035 | 0 | 0 | 0 |
| 13 | F4-R8 | 662 | 10601 | 11764 | 22365 |
| 14 | F4-R8 | 729 | 2798 | 3444 | 6242 |
| 15 | F3-R1 | 1871 | 2222 | 10599 | 12821 |
| 16 | F4-R8 | 576 | 170 | 186 | 356 |
| 17 | F4-R8 | 643 | 27 | 35 | 62 |
| 18 | F3-R1 | 1785 | 194 | 480 | 674 |
| 19 | F6-R8 | 816 | 13600 | 16684 | 30284 |
| 20 | F6-R8 | 883 | 2947 | 3495 | 6442 |
| 21 (F6) | F6-R1 | 2029 | 1571 | 8491 | 10062 |
| 21 (F5) | F5-R1 | 2022 | 1323 | 7397 | 8720 |
| 22 | F6-R8 | 730 | 1400 | 1697 | 3097 |
| 23 | F6-R8 | 797 | 801 | 905 | 1706 |
| 24 (F6) | F6-R1 | 1943 | 483 | 2347 | 2830 |
| 24 (F5) | F5-R1 | 1936 | 247 | 1120 | 1367 |
| **TOTAL:** | | | **56977** | **97677** | **154654** |
