# Supplementary material for: Alternative Isoform Analysis of Ttc8 Expression in the Rat Pineal Gland Using a Multi-Platform Sequencing Approach Reveals Neural Regulation
Source: PLoS One. 2016 Sep 29;11(9):e0163590. doi: 10.1371/journal.pone.0163590 (PMC5042479; doi:10.1371/journal.pone.0163590)
Supplement: S5 Table — See also Fig 6B. (DOCX) [file pone.0163590.s027.docx]

S5 table: Read-of-Insert counts for predicted potential isoforms with an added intron retention between exons 8 and 9b. See also Fig 6b.

| **Isoform**  **ID** | **Primer**  **Pair** | **Length** | **# Perfect Match** | **# Align**  **Match** | **Total**  **Match** |
| --- | --- | --- | --- | --- | --- |
| 02-IR | F1-R8 | 1930 | 322 | 950 | 1272 |
| 05-IR | F1-R8 | 1844 | 62 | 254 | 316 |
| 08-IR | F1-R8 | 1900 | 69 | 297 | 366 |
| 11-IR | F1-R8 | 1814 | 35 | 82 | 117 |
| 14-IR | F4-R8 | 1654 | 1148 | 3521 | 4669 |
| 17-IR | F4-R8 | 1568 | 277 | 722 | 999 |
| 20-IR | F6-R8 | 1808 | 2850 | 11179 | 14029 |
| 23-IR | F6-R8 | 1722 | 526 | 1770 | 2296 |
| TOTAL: | | | 5289 | 18775 | 24064 |
