# Supplementary material for: Alternative Isoform Analysis of Ttc8 Expression in the Rat Pineal Gland Using a Multi-Platform Sequencing Approach Reveals Neural Regulation
Source: PLoS One. 2016 Sep 29;11(9):e0163590. doi: 10.1371/journal.pone.0163590 (PMC5042479; doi:10.1371/journal.pone.0163590)
Supplement: S6 Table — See also Fig 3. Note that these counts are the number of ROI that perfectly match any of the theoretical potential isoforms that includes the given sub-variant. For example the first row (late donor, exon 4) compiles the counts from 8 different isoforms, all of which contain the late donor alternative splice junction on exon 4, as seen in Fig 6C. (DOCX) [file pone.0163590.s028.docx]

S6 table: Read-of-Insert counts for various extremely-low-coverage sub-variants. See also Fig 3. Note that these counts are the number of ROI that perfectly match any of the theoretical potential isoforms that includes the given sub-variant. For example the first row (late donor, exon 4) compiles the counts from 8 different isoforms, all of which contain the late donor alternative splice junction on exon 4, as seen in Fig 5c.

| **Sub-variant**  **Description** | **# Belonging to Perfect Match** | **# Belonging to Align**  **Match** | **Total Belonging to Match** |
| --- | --- | --- | --- |
| Late donor, exon 4 (exon 4a) | 263 | 632 | 895 |
| Skip exon 5 | 0 | 0 | 0 |
| Include 7a | 0 | 0 | 0 |
| Extra cassette exon 10a | 145 | 478 | 623 |
| Extra cassette exon 11a | 50 | 192 | 242 |
| Early acceptor, exon 12 (exon 12a) | 0 | 0 | 0 |
| Skip exon 13, end with exon 14a | 0 | 1 | 1 |
| **Totals:** | **458** | **1303** | **1761** |
